# Molecular and cellular characterization of four putative nucleotide transporters from the shrimp microsporidian *Enterocytozoon hepatopenaei* (EHP)

**DOI:** 10.1038/s41598-023-47114-8

**Published:** 2023-11-16

**Authors:** Orawan Thepmanee, Natthinee Munkongwongsiri, Anuphap Prachumwat, Vanvimon Saksmerprome, Sarocha Jitrakorn, Kallaya Sritunyalucksana, Rapeepun Vanichviriyakit, Sittinan Chanarat, Pattana Jaroenlak, Ornchuma Itsathitphaisarn

**Affiliations:** 1 Center of Excellence for Shrimp Molecular Biology and Biotechnology (Centex Shrimp), Faculty of Science, Mahidol University, Rama VI Rd., Bangkok, 10400 Thailand; 2 Department of Biochemistry, Faculty of Science, Mahidol University, Rama VI Rd., Bangkok, 10400 Thailand; 3 National Center for Genetic Engineering and Biotechnology (BIOTEC), National Science and Technology Development Agency (NSTDA), Yothi Office, Rama VI Rd., Bangkok, 10400 Thailand; 4 National Center for Genetic Engineering and Biotechnology (BIOTEC), National Science and Technology Development Agency (NSTDA), Thailand Science Park, Phahonyothin Rd., Pathum Thani, Klong Neung, Klong Luang 12120 Thailand; 5 Department of Anatomy, Faculty of Science, Mahidol University, Rama VI Rd., Bangkok, 10400 Thailand; 6 Laboratory of Molecular Cell Biology, Center for Excellence in Protein and Enzyme Technology, Faculty of Science, Mahidol University, Rama VI Rd. , Bangkok, 10400 Thailand; 7 Center of Excellence for Molecular Biology and Genomics of Shrimp, Department of Biochemistry, Faculty of Science, Chulalongkorn University, Phayathai Rd., Bangkok, 10330 Thailand

**Keywords:** Biochemistry, Gene expression, Microbiology, Fungi, Fungal biology

## Abstract

Microsporidia are obligate intracellular parasites that lost several enzymes required in energy production. The expansion of transporter families in these organisms enables them to hijack ATP from hosts. In this study, nucleotide transporters of the microsporidian *Enterocytozoon hepatopenaei* (EHP), which causes slow growth in economically valuable *Penaeus* shrimp, were characterized. Analysis of the EHP genome suggested the presence of four putative nucleotide transporter genes, namely EhNTT1, EhNTT2, EhNTT3, and EhNTT4. Sequence alignment revealed four charged amino acids that are conserved in previously characterized nucleotide transporters. Phylogenetic analysis suggested that EhNTT1, 3, and 4 were derived from one horizontal gene transfer event, which was independent from that of EhNTT2. Localization of EhNTT1 and EhNTT2 using immunofluorescence analysis revealed positive signals within the envelope of developing plasmodia and on mature spores. Knockdown of EhNTT2 by double administration of sequence specific double-stranded RNA resulted in a significant reduction in EHP copy numbers, suggesting that EhNTT2 is crucial for EHP replication in shrimp. Taken together, the insight into the roles of NTTs in microsporidian proliferation can provide the biological basis for the development of alternative control strategies for microsporidian infection in shrimp.

## Introduction

Microsporidia are obligate intracellular parasites. Inside host cells, proliferating microsporidia elevate the ATP demand for biosynthesis of macromolecules and interconversion of metabolites required in growth and differentiation^1^. Characteristically, microsporidia are known for their reduced genomes^2–4^, loss of glycolytic genes^5^, and nonfunctional mitochondria^6^ which, altogether, lead to an impaired oxidative phosphorylation^5^ and ultimately a restricted capacity for ATP production^1, 5, 7^.

In various microsporidian species, a group of membrane transporters namely nucleotide transporters (NTTs), ATP translocases (TLCs), or ATP/ADP carriers (AACs) serves to hijack host ATP^8–10^. A similar circumstance occurs in intracellular bacteria, such as Chlamydiae^11–13^ and Rickettsiae^14, 15^, where bacterial NTT-related proteins appropriate host ATP to support their own progression and development. Thus, it has been hypothesized that microsporidia acquired this group of genes by a lateral gene transfer from Chlamydiae and Rickettsiae^16–18^.

Four paralogous NTTs from human-infecting microsporidian species, namely *Trachipleistophora hominis*^8^ and *Encephalitozoon cuniculi*^9^ have been shown to transport host ATP into the parasites. Apart from ATP, microsporidian NTTs have also been shown to transport other purine nucleotides^8–10^. An NTT-related protein from the grasshopper-infecting microsporidian *Antonospora locustae*^10^, and four NTTs from *Enc. cuniculi*^9^ have been demonstrated to shuttle both ADP and ATP. Meanwhile, all of the *T. hominis* NTTs have been shown to transport ATP, ADP, GTP, and GDP^8^.

Localization assays demonstrated that the four paralogous NTTs from *T. hominis*^8^ are located on the plasma membrane of intracellular plasmodial stages where they can transport ATP and other nucleotides at the host-parasite interface. Surprisingly, the other *Enc. cuniculi* NTT^19^ and a heterologously-expressed NTT-like protein from *A. locustae*^10^ are colocalized to functionally reduced mitochondria or mitosomes, suggesting that their responsibility is to supply ATP for mitosomes.

*Enterocytozoon hepatopenaei* (EHP) is a microsporidian species that infects economically valuable penaeid shrimp in many countries of Asia^20–24^ and some areas of South America^25^. Three known species of shrimp, including *Penaeus vannamei*^21, 25, 26^, *P. monodon*^27^, and *P. stylirostris*^20^, and one suspected *P. japonicus*^28^, are infected with EHP. The infection causes stunted growth and size variation, resulting in economic losses^29–31^.

The genome of EHP contains four genes encoding nucleotide transporters (EhNTTs)^5^. However, sequence analysis, localization, and functions of the proteins on proliferation and pathogenicity of EHP are still poorly understood. In this study, we characterized the four EhNTTs in terms of their transcriptional expression levels, phylogeny, secondary structures, and localization in both extracellular and intracellular life stages. In addition, the requirement of one of the four EhNTTs in EHP infection was investigated using RNA interference (RNAi) technique. These findings will improve our knowledge on EHP biology and pathogenicity and ultimately lead to a development of effective approaches to control EHP infection.

## Results

### The genome of EHP contains four putative nucleotide transporters (EhNTTs)

The nucleotide and amino acid sequences of the four EhNTTs were analyzed (Table 1). The coding sequences of the four EhNTT genes are between 1668 and 1803 bp in size and encode 555–600 amino acid-long proteins. The transporters have approximated molecular weights of 64.2–69.4 kDa and predicted isoelectric point (pI) of 8.21–9.54. From an amino acid sequence alignment, the four EhNTTs share 20–34% identity (Supplementary Fig. S1).

**Table 1:** Biological properties of EhNTTs: coding sequence (CDS) lengths, primary sequence lengths, molecular weights, and predicted isoelectric points (pI).

Protein name	CDS length (bp)	Primary sequence length (aa)	Molecular weight (kDa)	Predicted pI
EhNTT1	1707	568	65	9.41
EhNTT2	1668	555	64.2	8.21
EhNTT3	1698	565	67	9.54
EhNTT4	1803	600	69.4	9.35

**Figure 1:**
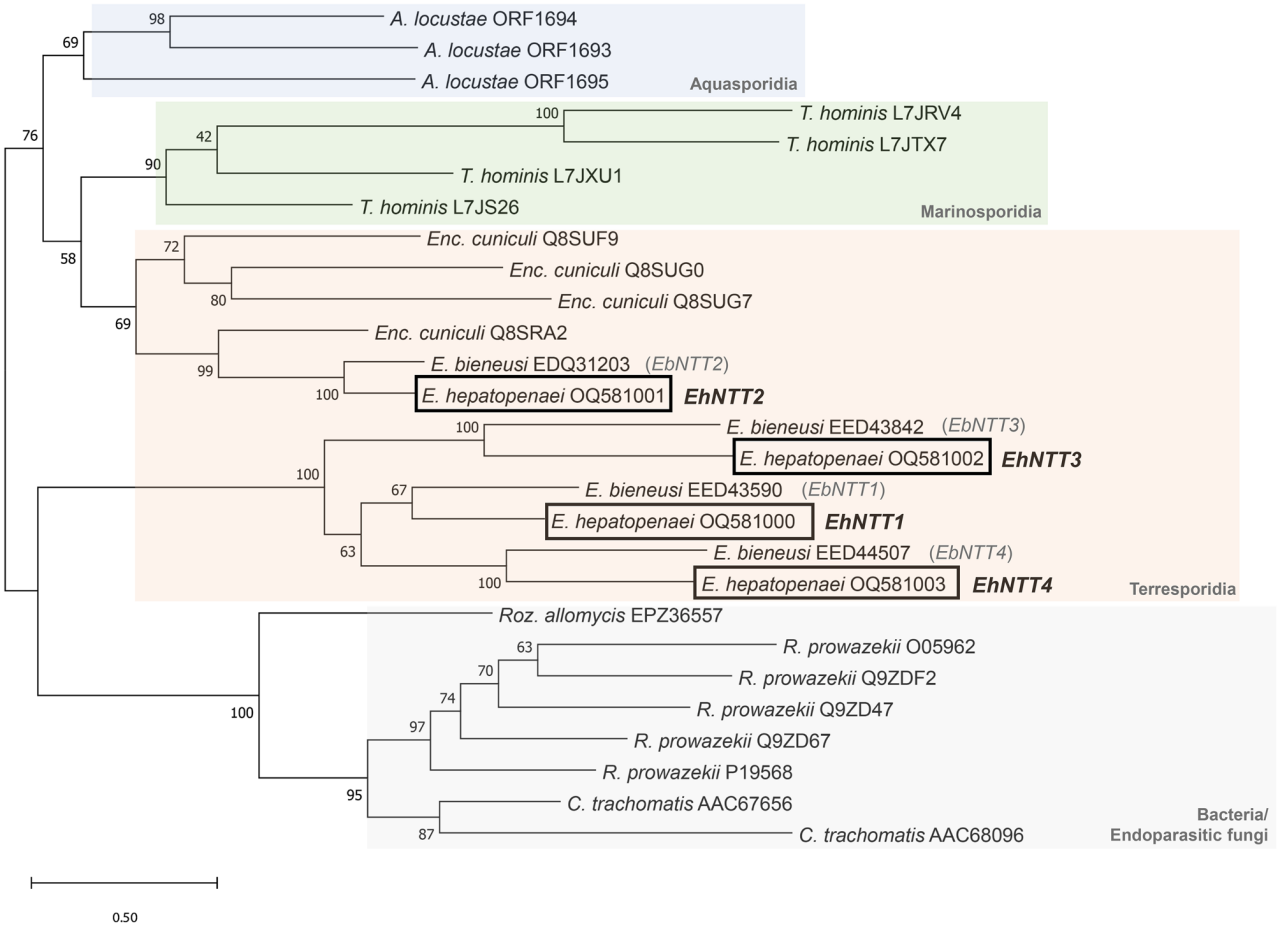
Phylogenetic analysis indicated that EhNTT1, 3, and 4 were derived from a single horizontal gene transfer event which was separated from that of EhNTT2. The phylogenetic tree was constructed using Maximum Likelihood method (1000 bootstrap replications). Bootstrap values are specified at each node.

To investigate the evolution of EhNTTs relative to those from other microsporidian species, the fungal endoparasite *Rozella allomyces*, and intracellular bacteria, a phylogenetic tree was constructed (Fig. 1). EhNTTs shared a common ancestor with NTTs from *E. bieneusi* and* Enc. cuniculi* which are members of the Apansporoblastina suborder. Among the four paralogs in EHP, EhNTT1, 3, and 4 belong to one clade, whereas EhNTT2 falls into the other clade with its orthologs from *E. bieneusi* and* Enc. cuniculi*.

**Figure 2:**
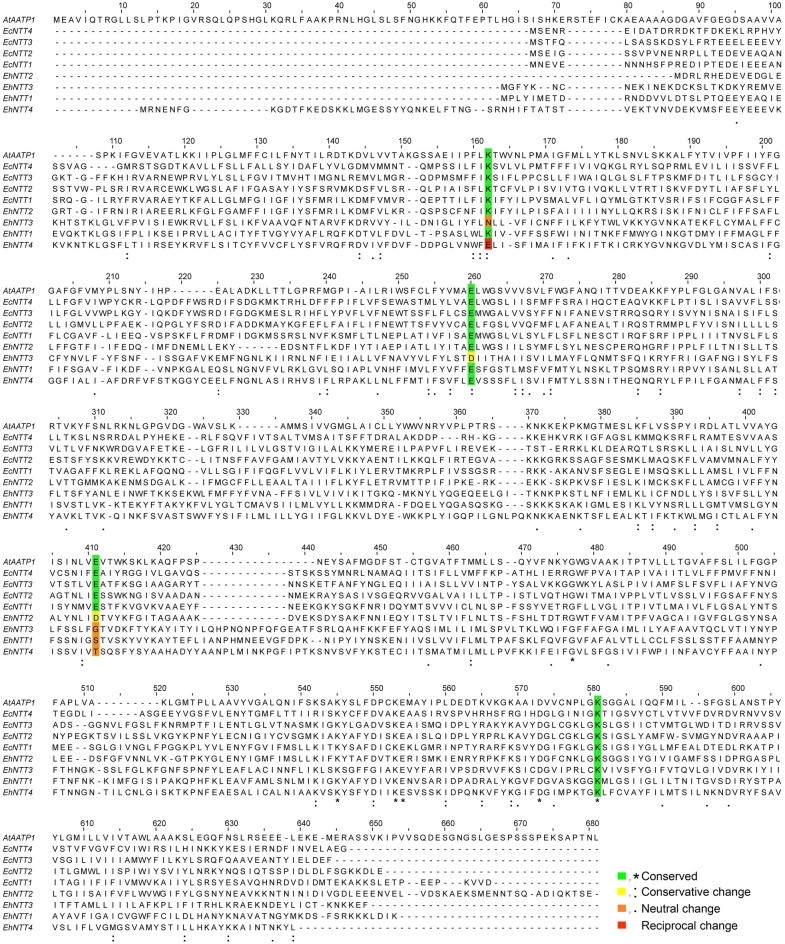
Sequence alignment showed four functionally conserved residues in *A. thaliana* (AtAATP1), *Enc. cuniculi* (EcNTT1-4), and EHP (EhNTT1-4). The four key residues (K155, E245, E385, and K527) of AtAATP1^32^ are highlighted according to their conservations. The multiple sequence alignment was constructed using the Clustal Omega server^33^.

There are four conserved amino acids that are functionally important for nucleotide transports in NTTs^32^. These residues are the most conserved in EhNTT2, followed by EhNTT1, 3, and 4, respectively (Fig. 2). The four charged residues K155, E245, E385, and K527 of plastidic ADP/ATP transporter 1 from *Arabidopsis thaliana* (AtAATP1)^32^ have been reported to be strongly conserved among NTTs from intracellular bacteria, the endoparasitic fungi *Roz. allomyces*, and microsporidian species^8, 9^. Amino acid sequence alignment of NTTs from *A. thaliana* (AtAATP1), *Enc. cuniculi* (EcNTT1-4), and EHP (EhNTT1-4) is shown in Fig. 2. The conservative features of the four conserved residues in amino acid sequences of EhNTTs were summarized in Table 2. Among the four conserved residues, K527 is the most conserved residue, followed by E245, K155, and E385, respectively (Fig. 2). A detailed multiple sequence alignment of NTT proteins from several organisms are shown in Supplementary Fig. S2.

**Table 2:** The four functionally important amino acid residues in *Arabidopsis thaliana* ADP/ATP transporter 1 (AtAATP1) and EhNTTs.

Conserved residues in AtAATP1	Changes of the key residues in EhNTTs							
	EhNTT1		EhNTT2		EhNTT3		EhNTT4	
	Aligned residues	Types of change	Aligned residues	Types of change	Aligned residues	Types of change	Aligned residues	Types of change
K155	K	Conserved	K	Conserved	N	Neutral	E	Reciprocal
E245	E	Conserved	E	Conserved	D	Conservative	E	Conserved
E385	S	Neutral	D	Conservative	G	Neutral	T	Neutral
K527	K	Conserved	K	Conserved	K	Conserved	K	Conserved

The secondary structures of EhNTT1-4 contain 10–12 α-helical transmembrane helices, which are typical of microsporidian NTTs^8, 9^ (Supplementary Figs. S3 and S4). The positions of the putative transmembrane helices in the four EHP paralogs are considerably conserved (Supplementary Fig. S2). As the four signature residues of NTTs are the most conserved in EhNTT2 (Fig. 2), the putative secondary structure of EhNTT2 was compared to those of other microsporidian species and intracellular bacteria. The comparison demonstrated similar positions of their transmembrane domains (Supplementary Fig. S2).

**Figure 3:**
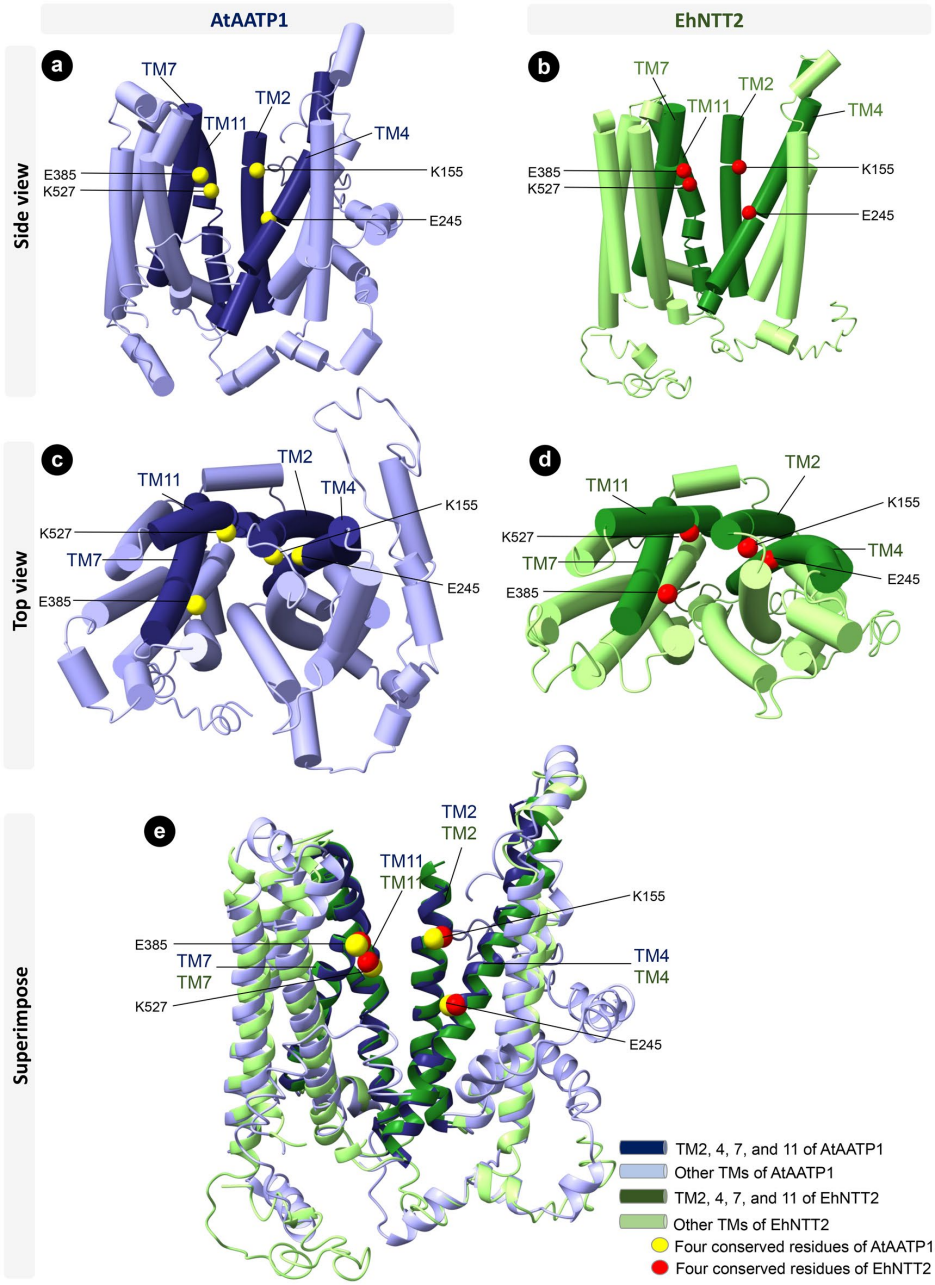
Predicted tertiary structures of AtAATP1 and EhNTT2 are comparable. The predicted tertiary structures of AtAATP1 (blue) and EhNTT2 (green) are depicted as a side view (**a**, **b**) and a top view (**c**, **d**). The putative transmembrane helices are shown as cylinders in a-d and ribbons in e. The four conserved residues of NTTs in the structure of AtAATP1 and EhNTT2 are shown as yellow and red spheres, respectively. The putative transmembrane helices of AtAATP1 and EhNTT2 which contain the four conserved residues are colored darker blue and green, respectively. Superimposition of the alpha carbons of the two structures (**e**) shows similar architectures. The three-dimensional structures were constructed using ChimeraX^35^.

To determine whether the four charged residues can potentially be involved in ATP transport, we predicted the tertiary structure of AtAATP1, the best biochemically characterized member of NTTs^32^, by the I-TASSER server^34^. The predicted structure of AtAATP1 revealed that the aforementioned charged residues reside along the pore-lining conduit (Fig. 3), hence implying their roles in ATP transport. As these putative residues are the most conserved in EhNTT2 (Fig. 2), the tertiary structure of EhNTT2 was predicted and compared to that of AtAATP1. By superimposing their alpha carbons, the alignment shows that the residues in the two transporters occupy similar positions within the pore-lining tracks (Fig. 3). This leads us to propose that the conserved residues in EhNTT2 may be involved in ATP transport.

### All four EhNTT genes were upregulated during cohabitation

**Figure 4:**
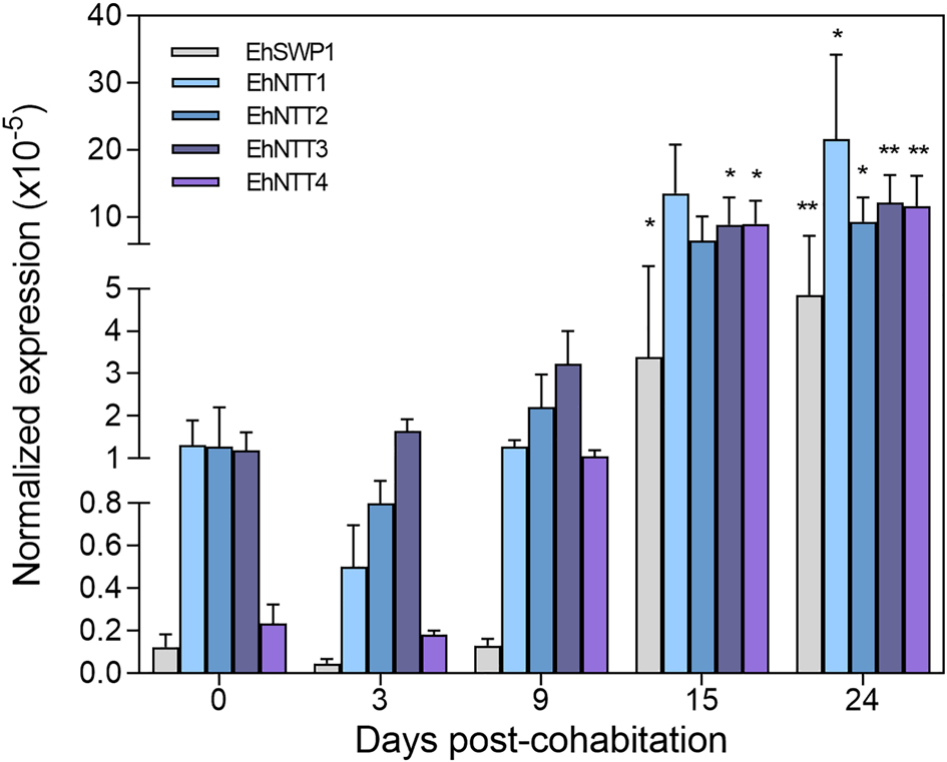
Four EhNTT genes were upregulated during cohabitation. At each day post-cohabitation, the copy numbers of EhNTT1, 2, 3, 4, and EhSWP1 genes were divided by the copy numbers of the Pv18srRNA gene and multiplied by 10^5^. The vertical bar graph represents the mean ± standard deviation of three replicates (except for the day 3 of cohabitation, which n = 2). The asterisks indicate significant difference of the normalized expression values of a gene (× 10^5^) at particular time point compared to those on day 0. For statistical analysis, the one-way ANOVA was applied. (* = *p* < 0.05, ** = *p* < 0.01).

To investigate the role of EhNTTs during EHP infection, the transcriptional levels of the four genes were quantified. Naïve shrimp were exposed to EHP by cohabitation^36^ before cDNA collection for RT-qPCR analysis. The level of the spore wall protein 1-encoding gene (EhSWP1) was used as a marker of infection, while the 18srRNA gene of *P. vannamei* (Pv18srRNA gene) was used as an internal control. Since the copy numbers of the Pv18srRNA gene were high, the normalized expression values were smaller than 1. By comparing the normalized gene expression levels before cohabitation, the EhSWP1, EhNTT3, and EhNTT4 genes were significantly upregulated after 15 days and maintained until day 24 (Fig. 4). At which point, all four EhNTT genes and the EhSWP1 gene were significantly upregulated compared to the starting day of cohabitation (Fig. 4). The elevation of EhNTT gene expression was possibly due to the replication of EHP inside the host cells.

### Polyclonal antibodies against EhNTT1 and EhNTT2 proteins are specific to their target fusion proteins

**Figure 5:**
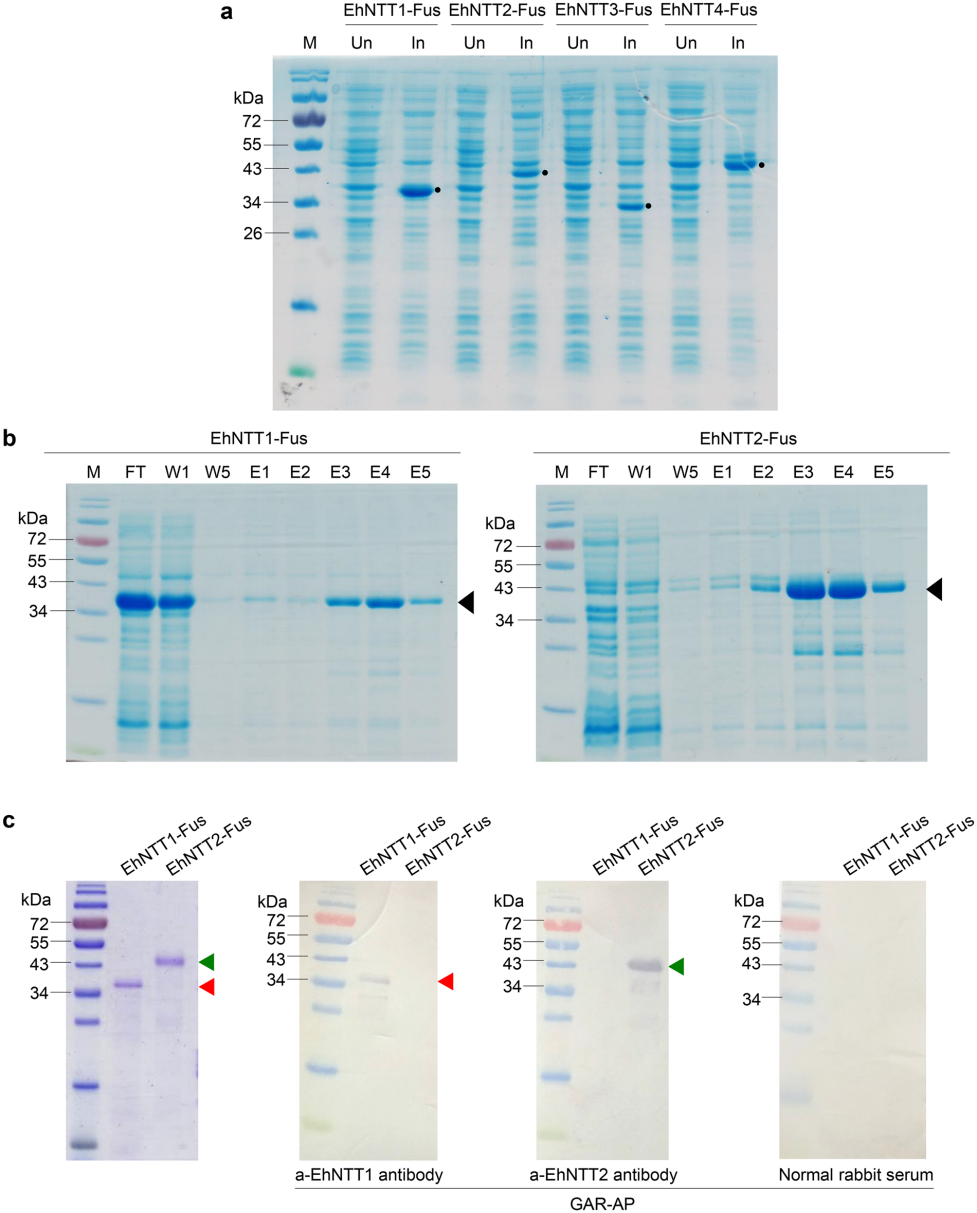
The recombinant fusion proteins EhNTT1 and 2 (EhNTT1-Fus and EhNTT2-Fus) were successfully expressed and purified, and the anti-EhNTT1 antibody and anti-EhNTT2 antibody effectively bind to their corresponding proteins without cross-reactivity. (**a**) Recombinant protein expression of four EhNTT fusion proteins in the *E. coli* strain BL21 star (DE3). Black dots highlight the overexpressed bands of each recombinant fusion protein. (**b**) Purification of EhNTT1-Fus (left) and EhNTT2-Fus (right) by Ni–NTA affinity chromatography. The expected bands of EhNTT1-Fus and EhNTT2-Fus were specified with red and green arrowheads, respectively. (**c**) Specificity tests of the anti-EhNTT1 antibody and anti-EhNTT2 antibody to their respective recombinant fusion proteins by western blot analysis. Normal rabbit IgG was used as a negative control. (Un, uninduced *E. coli* cells; In, induced *E. coli* cells; FT, flow-through fractions; W, wash fractions; E, elute fractions; GAR-AP, goat anti-rabbit antibody conjugated with alkaline phosphatase). Original blots/gels are presented in Supplementary Fig. S5.

To investigate the localization of EhNTTs in spores (an extracellular stage) and plasmodia (intracellular stages), a polyclonal antibody against each EhNTT was needed. The solvent-exposed regions of each EhNTT were combined to produce recombinant fusion proteins: EhNTT1-Fus, EhNTT2-Fus, EhNTT3-Fus, and EhNTT4-Fus. All four fusion proteins were successfully produced in *Escherichia coli* (Fig. 5a). Among the four proteins, EhNTT1-Fus and EhNTT2-Fus were successfully expressed and purified (Fig. 5b) for antibody production. Western blot assay showed that the anti-EhNTT1 antibody and anti-EhNTT2 antibody specifically detect their respective proteins with no cross-reactivity to the other (Fig. 5c). Hence, these two antibodies are suitable for further localization assays.

### EhNTT1 and EhNTT2 proteins are localized on the coat of the EHP spores and within the developing plasmodia

**Figure 6:**
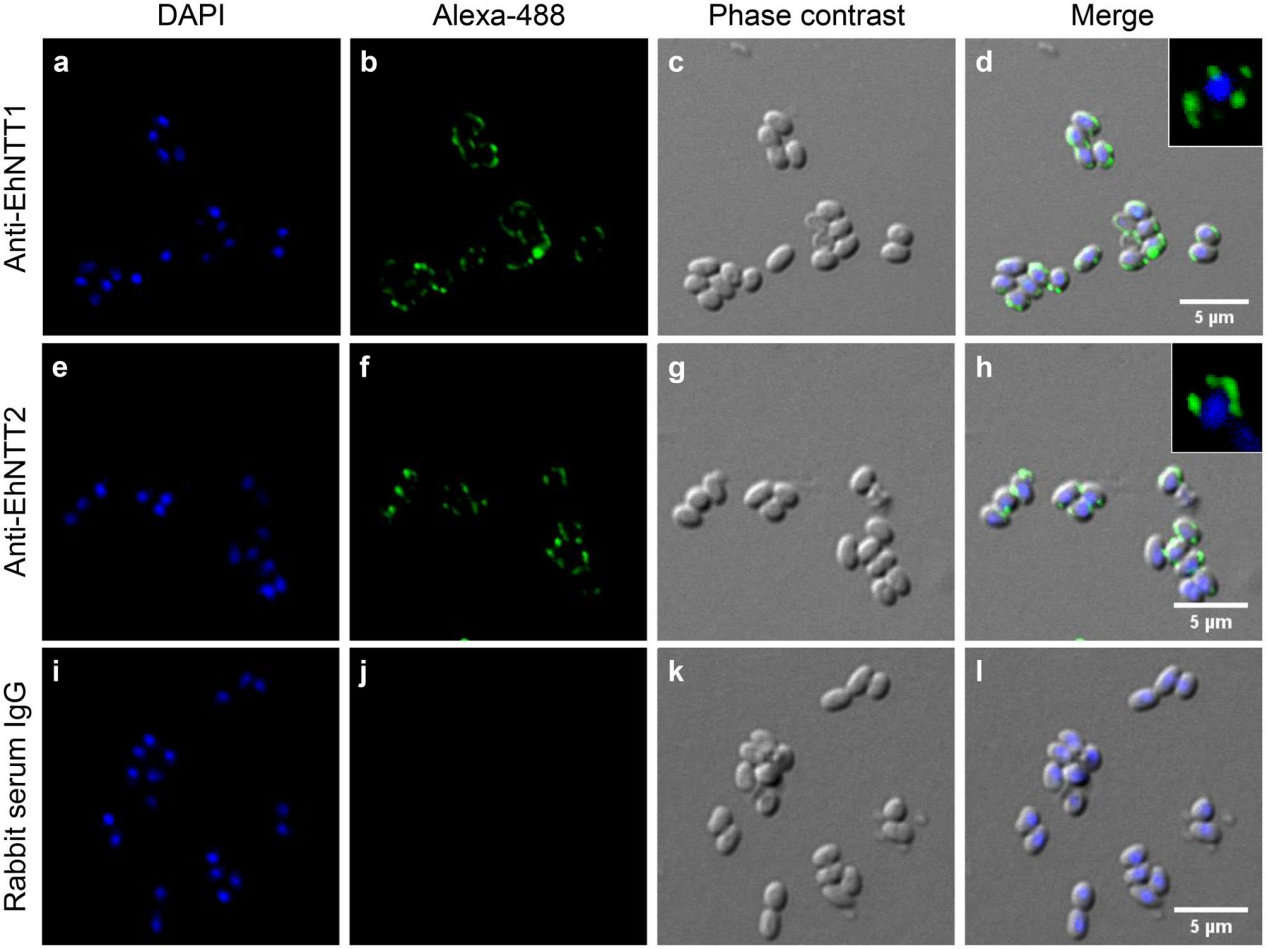
EhNTT1 and 2 were mainly localized on the spore coat. Localization of EhNTT1 and 2 in the isolated, mature EHP spores was performed using immunofluorescence analysis. (**a**, **e**, **i**) spore nuclei were stained with DAPI (blue). Rabbit antibodies against EhNTT1 (**b**) or EhNTT2 (**f**) indicated protein localization on the EHP spore coat (green). Normal rabbit IgG (**j**) was used as a negative control. (**c**, **g**, **k**) phase contrast micrographs. (**d**, **h**, **l**) merged images of DAPI, Alexa-488, and phase contrast. Enlarged images of spore expressing green fluorescence signal of EhNTT1 (**d**) or EhNTT2 (**h**) were included. The scale bar is 5 μm.

To study the localization of EhNTT1 and 2 in each developmental stage of EHP, an immunofluorescence analysis (IFA) was performed on purified, mature EHP spores and on EHP-infected hepatopancreatic tissue. IFA of the spores showed positive signals of the two proteins, primarily on the spore coat. It is possible that the signals were either on the spore wall layers or the plasma membrane of the spores (Fig. 6).

**Figure 7:**
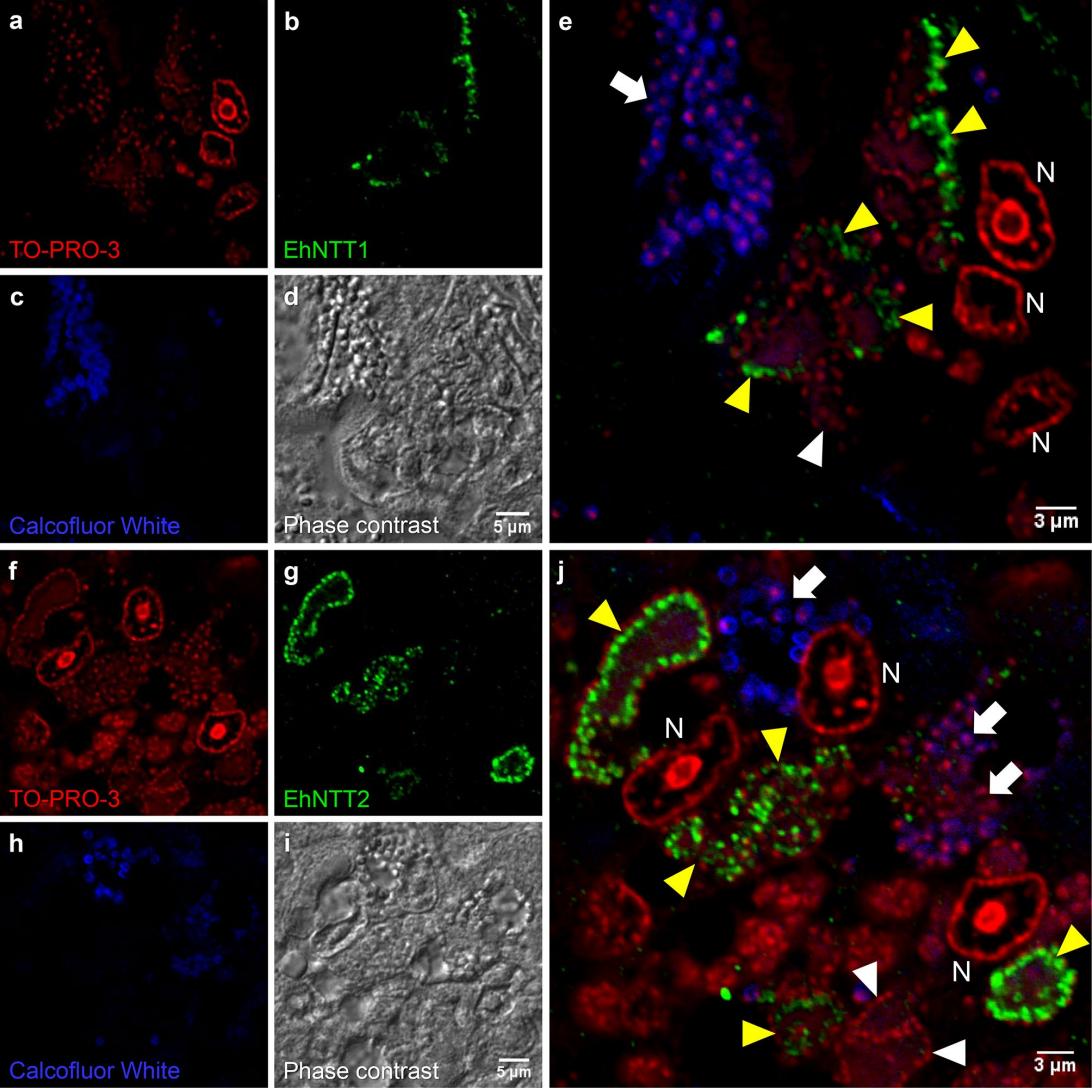
EhNTT1 and 2 were located within the plasma membrane of plasmodial stage replicating inside the infected shrimp hepatopancreatic tissue. Immunofluorescence analysis was performed with EHP-infected shrimp hepatopancreatic tissue. Red fluorescence labeled large nuclei of *P. vannamei* hepatopancreatic cells (N) and small nuclei of EHP in plasmodia and spore stages (**a** and **f**). Blue fluorescence labeled the chitin coat of mature spores (**c** and **h**). Green fluorescence labeled EhNTT1 (**b**) and EhNTT2 (**g**). Yellow arrow heads depict early sporogonial plasmodia while white arrow heads highlight late sporogonial plasmodia. (**d** and **i**, phase contrast images; **e** and **j**, overlaid images with three fluorescent channels). The phase contrast images merged with the three fluorescent channels are shown in Supplementary Fig. S6.

IFA of EHP-infected hepatopancreatic tissue of *P. vannamei* revealed that the signals of both EhNTTs were localized within the plasma membrane of sporogonial plasmodia (Fig. 7). Multi-nucleated sporogonial plasmodia are typical for the genus *Enterocytozoon*^37–39^. These sporogonial plasmodia are, in turn, found in vicinity to host nuclei, which is similar to *E. bieneusi*, the other microsporidian species of the genus^37, 38^. Within these plasmodia, EhNTT1 and 2 were either localized in proximity to peripherally-located nuclei of EHP or interspersed with nuclei of the parasite (Fig. 7). Nevertheless, some stages of the plasmodia showed negative signal of EhNTT1 and 2. It is suspected that these plasmodia might be late sporogonial plasmodia. Thicker membrane in these stages possibly results in reduced antibody accessibility^38, 39^.

In an effort to distinguish between sporogonial plasmodia and spores, the calcofluor white fluorescence dye was utilized. Calcofluor white is widely used to stain chitin on spore walls of microsporidian species^40–43^ and fungi^44, 45^. Even though both EhNTT1 and 2 were stained on the coat of the purified mature spores (Fig. 6), no signal were detected in spores differentiating within infected hepatopancreatic cells (Fig. 7). It is unclear why this is the case. One possible explanation might be an inability of the antibodies to penetrate extra-membranous layers presented in the host tissues and the thicker spore walls of the parasite^46, 47^.

### Knockdown of EhNTT2 reduces EHP replication in shrimp

**Figure 8:**
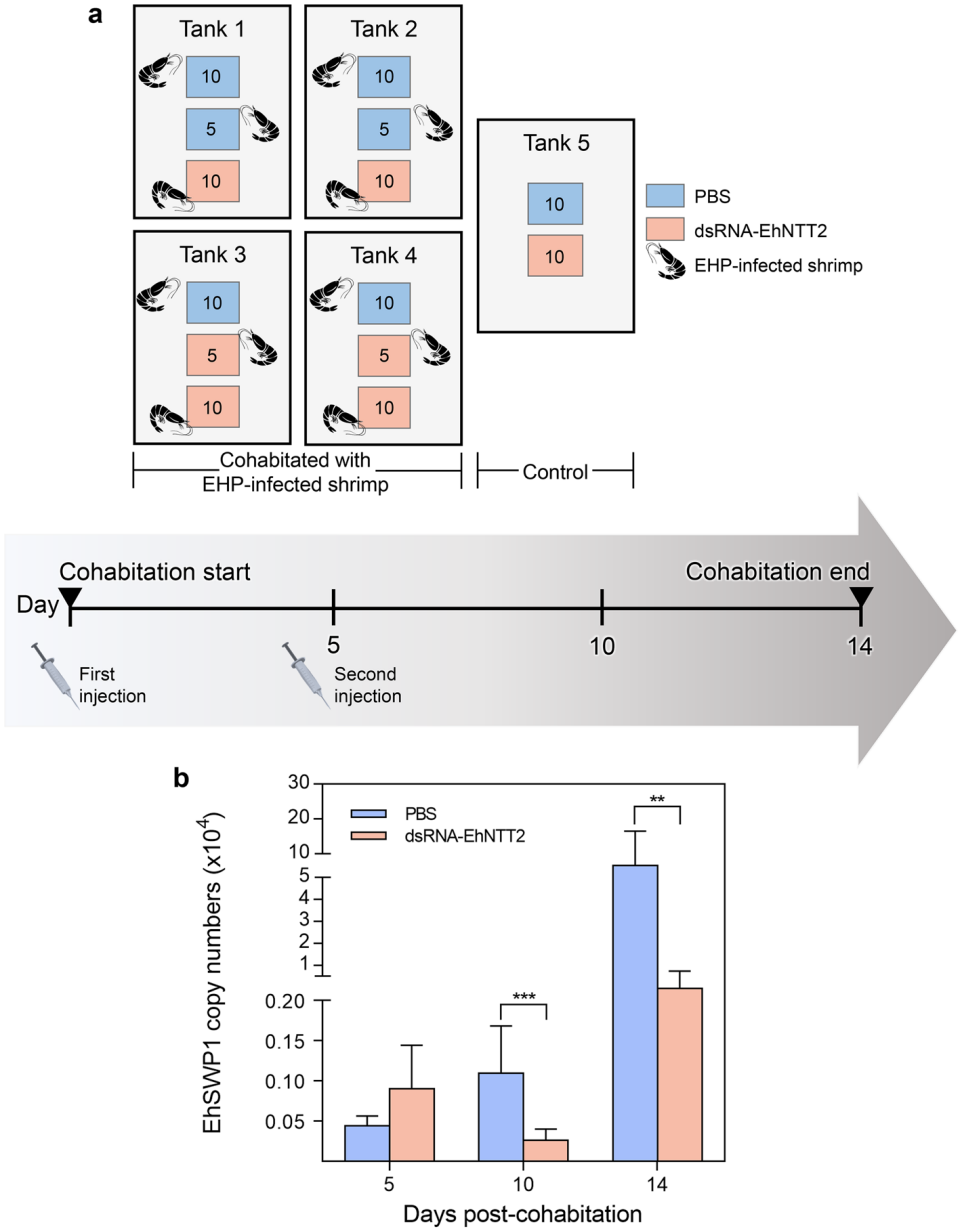
Knockdown of the EhNTT2 gene reduced EHP infection in shrimp. (**a**) A schematic diagram of the gene knockdown and cohabitation experiments. The cages containing PBS and dsRNA-EhNTT2 injected shrimp are shown as blue and pink boxes, respectively. The numbers of shrimp within each cage are specified. (**b**) The bar chart illustrating the mean ± standard deviation of copy numbers of the EhSWP1 gene per 100 ng of cDNA from the cohabitated shrimp collected on the day 5, 10, and 14 post-cohabitation, n = 10 per time point. The shrimp were doubly injected with PBS (blue bars) or dsRNA targeting the EhNTT2 gene (pink bars). The asterisks indicate the significant difference from the copy numbers of the EhSWP1 gene between the two groups. The significant levels were determined by the Mann–Whitney test. (** = *p* < 0.01, *** = *p* < 0.001).

To investigate an importance of EhNTT on EHP replication in shrimp, an RNAi assay was employed. As the phylogenetic analysis (Fig. 1) and the sequence alignment (Fig. 2 and Table 2) suggested that EhNTT2 is the most conserved among the four EhNTTs, it was selected as a candidate gene for the knockdown assay. The shrimp *P. vannamei* were intramuscularly injected twice with dsRNA targeting EhNTT2, prior to (day 0) and during cohabitation (day 5) with EHP-infected shrimp (Fig. 8a). Quantitative PCR result demonstrated that EHP infection levels were significantly reduced in the shrimp doubly administered with dsRNA-EhNTT2 (Fig. 8b). Comparing with the shrimp injected with PBS, those injected with dsRNA-EhNTT2 showed a significant decline in EHP copies after 10 days post-cohabitation, and the lower EHP infection level was observed until day 14 (Fig. 8b). This implicates that EhNTT2 is required for the proliferation of EHP in host cells.

## Discussion

### Phylogeny and amino acid sequence conservation of EhNTTs confirm that they are *bona fide* ATP transporters of EHP

Phylogenetic analysis suggested that putative NTTs from EHP share a common ancestor with NTTs from intracellular and other microsporidian species (Fig. 1). Previous studies proposed that the microsporidian species *T. hominis*^8^ and *Enc. cuniculi*^9^ acquired their NTTs from bacteria through a single gene transfer event, and subsequently duplicated into four paralogs. In this study, the phylogenetic analysis implies that the genus *Enterocytozoon* acquired these bacterial genes via two independent occasions. One transfer event provided NTT2, while the other yielded an ancestral gene of the other three NTTs. This ancestral gene could have undergone gene duplication which gave rise to the other three paralogs. The redundant NTT genes in the *Enterocytozoon* genomes underscores the energy dependence on host of the pathogens.

Four charged residues, including K155, E245, E385, and K527 are crucial for ATP transport in a plastidic ADP/ATP transporter from *A. thaliana* (AtAATP1)^32^. These four residues are widely conserved in NTTs from intracellular bacteria, the endoparasitic fungus *R. allomyces*, and most microsporidian species^8, 9^. In EHP, the four residues are the most conserved in EhNTT2, followed by EhNTT1, 4, and 3, respectively (Fig. 2). The comparison of the predicted tertiary structure of EhNTT2 and that of AtAATP1 reveals that these residues occupy equivalent positions within the pore-lining paths (Fig. 3). These findings suggest that EhNTT2 might possibly be an indispensable paralog. By gathering the results of the phylogenetic tree, signature sequence alignment, and tertiary structure comparison, we propose that the four NTTs are *bona fide* ATP transporters. Moreover, combination of these three analyses could serves as a general pipeline for identifying NTTs in other microsporidian species.

### EhNTTs are important for development of EHP

During EHP infection, the transcriptional levels of the four EhNTT genes increased in the infected shrimp (Fig. 4). The EhNTT3 and 4 genes were significantly upregulated before the other two paralogs (Fig. 4). This implies that the EhNTT3 and 4 genes might be required at the earlier stages of EHP proliferation, whereas the other two genes are possibly more involved in later development. The observation that the EhNTT genes are upregulated to the greater extent, when compared to the EhSWP1 gene, highlights the energy dependence on host of EHP.

The localization study in purified spores reveals that EhNTT1 and 2 are mainly localized on the spore coat (Fig. 6). This aligns with the previous proteomic results from EHP spore surface, which detected the presence of proteins related to nucleotide transport^48^. Corresponding to the evidence from other microsporidian species, their NTTs were detected in the spores^9, 49^. The reason why NTTs are present in microsporidian spores is still unknown. One possibility is that, during polar tube extrusion, these proteins are transported to host cells along with other infectious contents, so that the parasite can readily hijack host ATP to initiate proliferation. This hypothesis is supported by a previous immunofluorescence analysis of *Nosema bombycis* spores^50^. In this species, hexokinase-2 proteins are localized in both cytoplasm and on plasma membrane of the spores. Hexokinases phosphorylate hexose sugar at the top of the glycolytic pathway. Upon invasion, the fluorescent signals of hexokinase-2 were detected along the polar tubes. This result suggested that hexokinase-2 is directly transported into host cells. Another evidence supporting the direct transfer of sporoplasm proteins to host cells comes from electron microscopy which shows that spores are completely empty after germination^51^.

Although EhNTT1 and 2 were observed on the isolated spores, they were undetectable in the spores differentiating inside hepatopancreatic cells. The explanation for this observation is unclear. We propose that the inaccessibility of the intracellular epitopes might be due to the presence of connective tissues or additional membranes in the hepatopancreas. Another possibility is the effect of tissue fixation on antibody penetration as shown in previous study that the application of Davidson’s fixative instead of 4% paraformaldehyde produced less cytosolic signals^52^.

In addition to the extracellular spore stage, EhNTT1 and 2 were also detected in the intracellular plasmodial stages of EHP (Fig. 7)*.* The intracellular localization is consistent with former studies in other microsporidian species, including *T. hominis*^8^ and *Enc. cuniculi*^9^.

At the available resolution, EhNTT1 and 2 are not on the plasmodial membrane. Instead, they are localized in membranes of other sub-cellular compartments inside the plasmodia. Further experiments with higher resolutions are needed to narrow down the specific compartments of the two proteins.

### NTTs serve as potential targets for controlling microsporidian infection

Currently, many procedures, including washing of eggs and nauplii, cleaning the ponds with chemical such as calcium oxide (CaO) before stocking, and pasteurizing shrimp feed^31^ have been recommended to curb EHP spread. In this study, we propose an alternative approach for EHP control. Double injection of dsRNA-EhNTT2 in shrimp significantly reduced EHP replication (Fig. 8). Similarly, targeting NTTs with the RNAi technique in the honey bee-infecting microsporidian species^50^ and in the human-infecting protozoa^51, 52^ mitigate pathogen proliferation. The inhibitory effect of EhNTT2 silencing on EHP proliferation indicates that EhNTT2 is a potential target for EHP treatment. To further exploit the knockdown effect of RNAi in EHP control, a combination of dsRNAs targeting multiple EhNTTs should be investigated.

Nevertheless, the use of RNAi as a disease control measure is challenging, due to its cost at farm-scale application. Hence, rather than silencing microsporidian NTT transcripts, inhibiting the NTT proteins by using of small molecules could be more practical. To design those specific molecules, one important criterion is to avoid an off-target effect on hosts. In the EHP-penaeid shrimp system, the amino acid sequence similarity between four EhNTTs and NTT-related proteins, namely adenine nucleotide translocases, from *P. vannamei* and *P. monodon* is considerably low (11–19%; Supplementary Fig. S7). This might make it possible to design small molecules that exclusively target EhNTTs. A similar approach could, promisingly, be applied to manage microsporidian infection in human, since the degrees of amino acid sequence similarity between microsporidian NTTs and the NTT-like proteins of human are also low (12–20%; Supplementary Table S1). In addition, some has documented that microsporidian NTTs and NTT-related proteins from other animals, including human, are unrelated^9^. This supports the idea of using specific molecules to suppress the activity of the parasitic NTTs, with no side-effect on hosts. Further investigations are needed to develop these small molecules to control microsporidian infection in human, shrimp, and other organisms.

## Conclusion

In the present study, we characterized four nucleotide transporters from EHP (EhNTTs) in terms of their transcriptional expressions, localizations, and effects on EHP replication. The results showed that the four EhNTT-encoding genes were expressed at high levels during EHP infection. The EhNTT1 and 2 proteins were detected in both an intracellular plasmodial stage and an extracellular spore stage. Knocking down the EhNTT2 gene resulted in the decline of EHP replication. Altogether, these observations underpin crucial roles of EhNTTs as one of the virulent factors during EHP proliferation. Better understanding on such factors would lead to further development of strategies to suppress EHP infection in shrimp farms.

## Methods

### Primary sequence and structural analyses

To investigate the conservation of amino acid sequences of EhNTTs with NTTs from other microsporidian species and intracellular bacteria, a multiple sequence alignment was constructed using Clustal Omega^33^. The accession numbers of NTT sequences used in the alignment are shown in Supplementary Table S2. To study an evolutionary link between EhNTTs and NTTs from other species, phylogenetic analysis was performed using the maximum likelihood bootstrap method with MEGA (v11.3.11^53^). The three-dimensional structures of four EhNTTs were predicted by the I-TASSER protein structure and function prediction server (Supplementary Fig. S8)^34^. The resulted PDB files of EhNTT1 and EhNTT2 were submitted to PDBsum to generate schematic wiring diagrams of secondary structures of the two proteins (Supplementary Figs. S3 and S4)^54^. To designate structural features of EhNTTs including transmembrane helices, intracellular regions, and extracellular loops, their secondary structures were predicted by the Consensus Constrained TOPology prediction method (CCTOP^55^). Putative three-dimensional structures of proteins were constructed using ChimeraX^35^.

### Shrimp specimens and validation of EHP infection

The guidelines of the New South Wales State Government (Australia) for the human harvesting of fish and crustaceans were strictly followed during this research (https://www.dpi.nsw.gov.au/animals-and-livestock/animalwelfare/general/welfare-of-fish/shellfish). Naïve and EHP-infected *P. vannamei* were brought from different commercial shrimp farms in Thailand. Prior to laboratory induced infection assay, approximate 10% of the shrimp population were randomly selected for validation of EHP infection. Briefly, hepatopancreas (HP) of each shrimp was dissected. The genomic DNA was extracted using the QIAamp DNA Mini Kit (QIAGEN, Germany) following the manufacturer's instruction. The extracted DNA was used as template for SWP-PCR ^56^ to test for EHP infection prior to the beginning of downstream experiments.

### RNA extraction and cDNA synthesis for plasmid construction

To prepare complementary DNA (cDNA) template for recombinant plasmid construction, RNA was extracted from HP of the EHP-infected shrimp using the RiboZol RNA Extraction Reagent (VWR Life Science, USA) following the manufacturer’s protocol. Contaminated DNA was eliminated by treatment with RQ1 RNase-Free DNase (Promega, USA). Reverse transcription was performed to produce cDNA using the ImProm-II™ Reverse Transcription System (Promega, USA). An oligo dT was used as a primer and the protocol provided by the company was followed. The cDNA product was used as a template for production of recombinant plasmids, which were further employed for construction of the qPCR standard curves and production of dsRNA.

### Recombinant plasmid production for construction of qPCR standard curves and dsRNA production

To quantify the expression levels of each EhNTT gene by reverse transcription quantitative PCR (RT-qPCR), a qPCR standard curve is required. To construct the qPCR standard curves, recombinant plasmid containing an open reading frame (ORF) of each gene was produced. The complete ORFs of EhNTT1 (1707 bp), EhNTT2 (1668 bp), EhNTT3 (1698 bp), and EhNTT4 (1803 bp) were amplified from cDNA obtained from EHP-infected *P. vannamei* using primers listed in Table 3. The nucleotide sequences of EhNTT1-4 were deposited on the GenBank database with the accession numbers of OQ581000, OQ581001, OQ581002, and OQ581003, respectively. A 25 μl PCR reaction contained 100 ng cDNA template, 1 × Q5 reaction buffer, 0.2 mM dNTP, 0.2 µM forward primer, 0.2 µM reverse primer, and 0.5 unit of Q5 DNA polymerase (New England Biolabs, USA). The PCR conditions were used as follows: denaturation at 98 °C for 30 s followed by 35 cycles of 10 s denaturation at 98 °C, 30 s annealing at 55 °C, and 1 min extension at 72 °C, with a final extension for 1 min at 72 °C. Subsequently, PCR products were analyzed by 1.5% agarose gel electrophoresis to ensure expected amplicons. Then, the PCR products were purified using the QIAquick PCR Purification Kit (QIAGEN, Germany) prior to cloning into pET28a plasmids. The interested genes were inserted between *Nco*I and *Xho*I restriction sites, except for the full-length EhNTT2 gene which was cloned into a pBluescript SK( +) plasmid using an *EcoR*V restriction site. The recombinant plasmids generated in this experiment were pET28a_EhNTT1, pBluescript_EhNTT2, pET28a_EhNTT3, and pET28a_EhNTT4.

**Table 3:** List of primers used in this study.

Primer	Sequence (5′ → 3′)	References
**Recombinant plasmid construction**		
EhNTT1_FL_F_*Nco*I	ATATCCATGGCCATGCCCCTATATATA	This article
EhNTT1_FL_R_*Xho*I	ATATCTCGAGTTACTTAATATCAAGCTTC	
EhNTT2_FL_F_ *Nco*I	ATATCCATGGCC ATGGATAGACTAAGA	This article
EhNTT2_FL_R_*Xho*I	ATATCTCGAGTTATTCACTTGTTTTTTG	
EhNTT3_FL_F_*Nco*I	ATATCCATGGCCATGGGCTTTTAT	This article
EhNTT3_FL_R_*Xho*I	ATATCTCGAGTTAGAATTCTTTTTTATT	
EhNTT4_FL_F_*Nco*I	ATATCCATGGCCATGAGAAATGAA	This article
EhNTT4_FL_R_*Xho*I	ATATCTCGAGTTAAAGATATTTATTGGT	
EhSWP_FL_F_*Nco*I	ATATCCATGGGCATGTTAGAAGATGCAAAG	^57^
EhSWP_FL_R_*Xho*I	ATATCTCGAGAGAAAATTTTTCAAGGTG	
Pv18srRNA_qF	GAGACGGCTACCACATCTAAG	^58^
Pv18srRNA_qR	ATACGCTAGTGGAGCTGGA	
**Realtime PCR detection**		
EhNTT1_qF	CCATTCCTGCAAAACAGCCTC	This article
EhNTT1_qR	CCTGTCAGCCGGATCAATTC	
EhNTT2_qF	GGCTGGACATTTGTTGCGAT	This article
EhNTT2_qR	AATCTTCCTCAAGCGCCGAA	
EhNTT3_qF	TTTTTCACGATTGCAGGCACA	This article
EhNTT3_qR	CACTGTGTAACTGCCGCAAA	
EhNTT4_qF	GCATATTTTTGCGTCCGGCA	This article
EhNTT4_qR	ACAACGCCATATTAGCACCA	
EhSWP_1F	TTGCAGAGTGTTGTTAAGGGTTT	^56^
EhSWP_2R	GCTGTTTGTCTCCAACTGTATTTGA	
Pv18srRNA_qF	GAGACGGCTACCACATCTAAG	^58^
Pv18srRNA_qR	ATACGCTAGTGGAGCTGGA	
**Synthesis of dsRNA-EhNTT2**		
dsEhNTT2F-sense	TAATACGACTCACTATAG GAGACAAAGCCCTTCTTGTT	This article
dsEhNTT2R-sense	CTGTTAGGGCAGCTTCTAAC	
dsEhNTT2F-antisense	TAATACGACTCACTATAG CTGTTAGGGCAGCTTCTAAC	This article
dsEhNTT2R-antisense	GAGACAAAGCCCTTCTTGTT	

To quantify the EHP infection level, the mRNA expression of a spore wall protein 1-encoding gene (EhSWP1) was measured by RT-qPCR assay. It has been demonstrated that EhSWP1 appears as a single copy gene in the EHP genome^5^. The complete ORF of EhSWP1 gene (678 bp) was amplified from cDNA obtained from EHP-infected *P. vannamei* (GenBank accession no. MG015710) using primers listed in Table 3. The PCR protocol was the same as previously described in^56^. The PCR product was analyzed on an agarose gel and purified prior to cloning into a pGEM®-T Easy cloning vector (Promega, USA) using an *EcoR*I restriction site to generate a recombinant plasmid pGEM_EhSWP1.

An 18s rRNA gene of *P. vannamei* (Pv18srRNA gene) was used as an internal control for the RT-qPCR assay. A part of Pv18srRNA gene (204 bp) was amplified from cDNA obtained from a naive *P. vannamei* (GenBank accession no. XR_003477346) using primers specific to the 18s rRNA gene (Table 3). The same PCR protocol for amplification of EhSWP1 gene was used. The PCR product was purified and cloned into a pGEM®-T Easy cloning vector using an *EcoR*I restriction site to generate a recombinant plasmid pGEM_Pv18srRNA.

All six recombinant plasmids including pET28a_EhNTT1, pBluescript_EhNTT2, pET28a_EhNTT3, pET28a_EhNTT4, pGEM_EhSWP1, and pGEM_Pv18srRNA were transformed into the * E. coli* strain DH5α for plasmid propagation. The recombinant plasmids were extracted using the Presto™ Mini Plasmid Kit (Geneaid, Taiwan) following the manufacturer’s instruction. The positive clones were selected by restriction endonuclease analysis which were subsequently confirmed by DNA sequencing (Macrogen, South Korea). The recombinant plasmid pBluescript_EhNTT2 was further used as a template to produce of dsRNA targeting EhNTT2 gene, which were employed in the gene knockdown assay. Details of all the plasmids constructed in this study was summarized in Supplementary Table S3.

### Recombinant plasmid construction for the expression of fusion proteins

To study the localization of EhNTTs, putative solvent exposed regions of EhNTTs were selected and combined to generate fusion proteins. The putative solvent exposed regions were chosen from the secondary structure of each EhNTT as predicted from CCTOP and visualized by PDBsum (Supplementary Figs. S3 and S4)^54^. Combined nucleotide sequences of the solvent exposed loops were synthesized by commercial gene synthesis facility (Synbio Technologies, USA). Then, they were cloned into pUC57 cloning vectors, between *Nco*I and *Xho*I restriction sites, to generate the recombinant plasmid pUC57_EhNTT1_Fus, pUC57_EhNTT2_Fus, pUC57_EhNTT3_Fus, and pUC57_EhNTT4_Fus. The fusion gene inserts were further subcloned into a pET28a expression vector containing 6XHis tag at N-terminus. The obtained recombinant plasmids were named as pET28a_EhNTT1_Fus, pET28a_EhNTT2_Fus, pET28a_EhNTT3_Fus, and pET28a_EhNTT4_Fus.

### Expression and purification of recombinant fusion EhNTTs

Each of the recombinant plasmids pET28a_EhNTT1_Fus, pET28a_EhNTT2_Fus, pET28a_EhNTT3_Fus, and pET28a_EhNTT4_Fus with correct sequences was transformed into the *E. coli* strain BL21 star (DE3). The *E. coli* cells were grown in LB medium containing 50 μg/ml kanamycin and induced with 0.4 mM IPTG at 37 °C for 4 h. The cells were harvested by centrifugation at 17,700 × g at 4 °C for 10 min. Subsequently, all four recombinant EhNTT fusion proteins were purified by Ni–NTA affinity chromatography under a denaturing condition. Briefly, the bacterial cell pellet was resuspended with a lysis buffer (10 mM Tris, pH 8.0, 200 mM NaCl, 5% glycerol, and 8 M urea) in the presence of 1X protease inhibitor cocktail (Roche, Switzerland). The cells were sonicated on ice for 15 min followed by centrifuging at 17,700 × g, 4 °C for 10 min. The supernatant was collected and mixed with the lysis buffer. Then, the mixture was incubated with the Ni–NTA agarose beads (GE Healthcare, USA) at 4 °C for 1 h followed by loading onto a polypropylene column (QIAGEN, USA). The beads bound to the His-tagged fusion EhNTTs were washed with a wash buffer (10 mM Tris, pH 8.0, 200 mM NaCl, 5% glycerol, 20 mM imidazole, and 8 M urea). The recombinant EhNTT fusion proteins were eluted with an elution buffer (10 mM Tris, pH 8.0, 200 mM NaCl, 5% glycerol, 250 mM imidazole, and 8 M urea). The purified fusion protein EhNTT1-Fus, EhNTT2-Fus, EhNTT3-Fus, and EhNTT4-Fus were visualized on a 12.5% SDS-PAGE gel with expected molecular weight of 34, 36.6, 35.6, and 44 kDa, respectively. Among the four EhNTT fusion proteins, the recombinant EhNTT1 and 2 fusion proteins were produced with high quantity and acceptable purity. As a result, the EhNTT1 and 2 fusion proteins were used for the polyclonal antibody production. The purified recombinant fusion protein EhNTT1 and 2 were dialyzed, concentrated, and lyophilized before submitting them to a commercial antibody production unit.

### Antibody production and efficiency test

To produce polyclonal antibodies against either of the fusion protein EhNTT1 or 2, the purified fusion protein was immunized into two rabbits to produce rabbit anti-EhNTT1 antibody and anti-EhNTT2 antibody. The antibody production was performed by ChinaPeptides Co., Ltd., China.

An efficiency and cross-reactivity of both antibodies were tested by western blot analysis. Briefly, 500 ng of EhNTT1 and 2 fusion proteins were run on a 12.5% SDS-PAGE gel and then transferred onto a PVDF membrane. The membrane was washed with 1X PBS and incubated with a blocking solution (5% skimmed milk in 1X PBS) at RT for 1 h. For antibody binding reaction, 1:2000 anti-EhNTT1 or anti-EhNTT2 antibody in blocking solution was applied onto the membrane. A normal rabbit serum was used as a negative control. Each membrane was incubated with the primary antibody at 4 °C overnight followed by washing with PBST (0.05% Tween-20 in 1X PBS) for 3 times, 5 min each. Later, the membranes were immersed in a 1:2000 goat anti-rabbit antibody conjugated with alkaline phosphatase (GAR-AP) and incubated at RT for 1 h. After the washing steps, the membranes were incubated with an AP-substrate buffer (100 mM Tris–HCl, pH 9.5, 100 mM NaCl, 5 mM MgCl_2_) for 5 min and the signal was developed using NBT/BCIP solution (Merck Millipore Calbiochem, Germany).

### Cohabitation assay

To study the transcriptional levels of the four EhNTT genes, cohabitation assay was performed^36^. Three days before starting the experiment, 3–5 g naïve shrimp and 5–8 g EHP-infected shrimp were separately acclimated in aerated 20 ppt artificial sea water. The cohabitation assay was performed within two 500-L tanks, each containing 2/3 volumes of continuously aerated 20 ppt artificial sea water. In each tank, 45 naïve shrimp were cohabitated with 15 EHP-infected shrimp, which were separated by plastic cages. Two cages, each containing 7–8 EHP-infected shrimp, were put in each tank. Meanwhile, the naïve shrimp were put outside of the cages. The shrimp were cohabitated for 24 days. On 0, 3, 9, 15, and 24 days post-cohabitation, the shrimp HPs were collected for RNA extraction (3 shrimp per time point). Subsequently, the cDNA was synthesized and used as a template for RT-qPCR analysis.

### Reverse transcription quantitative polymerase chain reaction (RT-qPCR) analysis

To determine the mRNA expression levels of six interested genes including EhNTT1-4, EhSWP1, and Pv18srRNA, RT-qPCR was performed. The primers used in this assay are listed in Table 3. Each 20-μl reaction comprised 50 ng of cDNA template, 1 × KAPA SYBR® FAST qPCR Master Mix (Roche, Switzerland), 0.2 μM forward primer, and 0.2 μM reverse primer. The 2-step qPCR protocol started with an initial activation at 95 °C for 3 min followed by 40 cycles of denaturation at 95 °C for 3 min and annealing at 55 °C for 30 s. The copy numbers of each gene were calculated from standard curves which were constructed as described below. At each day post-cohabitation, the copy numbers of each gene were divided by the copy numbers of the Pv18srRNA gene and multiplied by 10^5^. The data was shown as normalized expression levels. Statistical analyses were performed using one-way ANOVA with Dunnett's multiple comparisons test.

To construct the qPCR standard curves, the recombinant plasmid pET28a_EhNTT1, pBluescript_EhNTT2, pET28a_EhNTT3, pET28a_EhNTT4, pGEM_EhSWP1, and pGEM_18srRNA were used as DNA templates at the concentration of 10^2^–10^8^ copies. The qPCR reactions were the same as those used for the mRNA expression level analysis. The qPCR standard curves for six interested genes as well as the qPCR efficiencies and coefficients of determination (R^2^) were shown in Supplementary Fig. S9.

### EHP spore purification

To prepare EHP spores for the immunolocalization assay, the spores were isolated from EHP-infected shrimp using the Percoll gradient centrifugation method, as previously described^59^. Briefly, HP from 10 EHP-infected shrimp were dissected and ground with a glass pestle. The ground tissue was mixed with distilled water and filtered with 100- and 40-µm filters, respectively. The filtrate was centrifuged at 4500 × g for 10 min at RT and the pellet was resuspended with distilled water. The mixture was loaded onto an ultracentrifuge tube containing a gradient of Percoll including 100%, 75%, 50%, and 25% Percoll (Cytiva, USA) which were diluted in 1X PBS. The tube was ultracentrifuged at 15,200 × g for 30 min at 15 °C. The mature EHP spores exhibiting the most germination rate (typically lied at the boundary between 75 and 100% Percoll solutions) were removed and washed with distilled water several times. The spores were resuspended with 1X PBS and stored at RT until use.

### Immunofluorescence analysis of EhNTT1 and EhNTT2 in purified EHP spores

To study the localization of EhNTT1 and 2 in the EHP spores, the purified spores were placed on glass slides coated with Histogrip (Thermo Fisher Scientific, USA) at the density of 10^5^ spores/slide and dried in a chemical hood at RT for 1 h. For spore fixation, the slides were incubated with 4% paraformaldehyde at RT for 20 min followed by washing with 1X PBS at RT for 3 times, 5 min each. Later, 1% Triton X-100 was added onto the slides and incubated for 30 min at RT to increase permeability of the spore. After washing steps, a blocking solution (10% normal goat-serum and 5% bovine serum albumin in 1X PBS) was added and incubated for 1 h. Then, 1:100 of either anti-EhNTT1 antibody or anti-EhNTT2 antibody in blocking solution was applied on the slides and incubated at 4 °C overnight. Normal rabbit IgG was used as a negative control. After the washing steps, 1:200 of goat anti-rabbit antibody conjugated with Alexa488 (Abcam, England) was added and the slides were incubated at RT for 1 h followed by washing steps. For staining of the host and EHP nuclei, the slides were incubated with 1:1000 DAPI staining solution (Abcam, England) in 1X PBS for 5 min followed by the washing steps. The slides were mounted with Prolong Gold Antifade Mountant (Invitrogen, USA) and visualized under the Olympus Fluoview FV1000i confocal laser scanning microscope (Center of Nanoimaging, Mahidol University).

### Immunofluorescence analysis of EhNTT1 and EhNTT2 in EHP-infected hepatopancreatic tissue

To investigate the presence of EhNTTs in intracellular stages of EHP, cephalothoraxes of EHP-infected shrimp were fixed with a Davidson’s fixative for 24 h, followed by a general shrimp histological process^60^. Paraffin-embedded tissue was sectioned and placed on poly-lysine coated glass slides (Bio Optica Milano S.p.A., Italy). After paraffin removal using xylene, the tissue was rehydrated with decreasing concentration of ethanol. For antigen retrieval, boiled 1X citrate buffer pH 6.0 containing 0.5% Tween-20 was added onto the slides and incubated at RT for 5 min, twice. The slides were washed twice with 1X PBST (0.05% Tween-20 in 1X PBS), 5 min for each, followed by permeabilizing with 0.4% Triton X-100 in 1X PBS for 5 min, twice. After blocking with 10% fetal bovine serum (FBS) in 1X PBS for 30 min, 1:100 of corresponding primary antibody or normal rabbit serum, diluted with 1%FBS, was applied onto the slides. The slides were incubated at 4 °C overnight. After the washing steps, 1:500 of goat anti-rabbit antibody conjugated with Alexa 488 (Abcam, England) in 1% FBS was added and the slides were incubated at RT for 1 h. The slides were washed twice before counterstaining with 1:500 of TO-PRO-3 nuclear staining dye (Thermo Fisher Scientific, USA) and 50 ng of Calcofluor White (Sigma-Aldrich, Germany) for 5 min at RT. After washing steps, the slides were mounted and visualized under Olympus Fluoview FV1000i confocal microscope.

### Production of dsRNA targeting EhNTT2 gene

The recombinant plasmid pBluescript_EhNTT2 was used as a template to generate target amplicons for an in vitro transcription reaction. The target amplicons for the production of sense and antisense ssRNAs of EhNTT2 gene were amplified by PCR using Green PCR Master Mix Direct-Load (Biotechrabbit, Germany). The PCR conditions were the same as those for the amplification of the EhSWP1 gene from shrimp cDNA. The primers of EhNTT2-sense and -antisense were designed with the T7-promoter sequence overhang at their 5′ end (underlined in Table 3). The purified PCR products were used to synthesize sense and antisense ssRNAs of EhNTT2 by in vitro transcription reaction using T7 RiboMAX™ Large Scale RNA Production Systems (Promega, USA). The manufacturer’s protocols were followed. After removing the leftover DNA template by DNase (Promega, USA), the sense and antisense ssRNAs of EhNTT2 were annealed using an annealing buffer (10 mM Tris–HCl, pH 8.0, 20 mM NaCl, 1 mM EDTA) at 70 °C for 15 min. The dsRNA products were verified by treatment with RNase A and RNase III. A 10 μl RNase A treatment reaction composed of 100 ng of dsRNA, 1X RNase A buffer (10 mM Tris–HCl, pH 8.0, 5 mM EDTA, 133 mM sodium acetate), and 50 ng of RNase A (Merck, Germany). A 10 μl RNase III treatment reaction included 100 ng of dsRNA, 1X ShortCut RNase III, 1X MnCl_2_, and 1.5 unit of ShortCut RNase III (Biolabs, England). Each of the reaction was incubated at 37 °C for 5 min and visualized on 1.5% agarose gel.

### Gene knockdown assay

Prior to starting an experiment, naïve shrimp (3–4 g) and EHP-infected shrimp (5–6 g) were acclimated in separate tanks filled with 25 ppt artificial sea water for three days. The naïve shrimp were divided into two groups (60 shrimp each group), including PBS (positive control) group and dsRNA-EhNTT2 (experimental) group, which were intramuscularly injected with 1X PBS and dsRNA against EhNTT2, respectively. On day 0, the naïve shrimp from the PBS group and the dsRNA-EhNTT2 group were injected with 1X PBS and 10 μg dsRNA/g shrimp of dsRNA-EhNTT2, respectively. Five or ten injected shrimp were placed in a cage separately for corresponding groups inside four 500-L tanks containing 2/3 volumes of 25 ppt artificial sea water. Each 500-L tank contained three cages of the injected shrimp and 30 EHP-infected shrimp outside of the cages (see arrangement of the cages in Fig. 8a). Similarly, a cage of 10 PBS shrimp and a cage of 10 dsRNA-EhNTT2 shrimp were placed in another 500-L tank without EHP-infected shrimp as a negative control group. After 5 days post-cohabitation, the remaining shrimp from the PBS group and the dsRNA-EhNTT2 group were injected again with 1X PBS and 5 μg of the dsRNA/g shrimp, respectively. The experiment was conducted until the day 14th after the first injection. Ten shrimp in the cohabitation tanks were collected randomly from the cages at 5, 10, and 14 days post-cohabitation, 10 shrimp per time point. Shrimp HP were dissected for DNA extraction and further subjected for the qPCR analysis.

To determine the EHP copy numbers in the shrimp obtained from the gene knockdown assay, qPCR of the EhSWP1 gene was performed using primers listed in Table 3. Each 20 μl qPCR reaction comprised of 10 ng of DNA template, 1X KAPA SYBR® FAST qPCR Master Mix Universal (Kapa Biosystems, South Africa), 1X ROX Low, 0.2 μM of forward primer, and 0.2 μM of reverse primer. The 3-step qPCR protocol started with an initial activation at 94 °C for 2 min followed by 40 cycles of denaturation at 94 °C for 15 s, annealing 60 °C for 30 s, and extension at 72 °C for 32 s. The copy numbers of EhSWP1 gene in the DNA samples were calculated using the standard curve previously constructed (Supplementary Fig. S9).

### Ethics approval and consent to participate

The guidelines of the New South Wales State Government (Australia) for the human harvesting of fish and crustaceans were strictly followed during this research (https://www.dpi.nsw.gov.au/animals-and-livestock/animalwelfare/general/welfare-of-fish/shellfish).

### Supplementary Information

Supplementary Figures.

Supplementary Table S1.

Supplementary Table S2.

Supplementary Table S3.

Supplementary Table S4.

## Data Availability

All data generated or analysed during this study are included in this published article (and its Supplementary Information files).
