# Supplementary material for: Molecular and cellular characterization of four putative nucleotide transporters from the shrimp microsporidian Enterocytozoon hepatopenaei (EHP)
Source: Sci Rep. 2023 Nov 16;13:20008. doi: 10.1038/s41598-023-47114-8 (PMC10654386; doi:10.1038/s41598-023-47114-8)
Supplement: Supplementary file 1 — Supplementary Figures. [file 41598_2023_47114_MOESM1_ESM.pdf]

Molecular and cellular characterization of four putative nucleotide transporters from the shrimp microsporidian *Enterocytozoon hepatopenaei* (EHP)

Orawan Thepmanee[a,b], Natthinee Munkongwongsiri[c], Anuphap Prachumwat[a,c], Vanvimon Saksmerprome[a,d], Sarocha Jitrakorn[a,d], Kallaya Sritunyalucksana[c], Rapeepun Vanichviriyakit[a,e], Sittinan Chanarat[b,f], Pattana Jaroenlak[g,*], Ornchuma Itsathitphaisarn[a,b,*]

[a]Center of Excellence for Shrimp Molecular Biology and Biotechnology (Centex Shrimp), Faculty of Science, Mahidol University, Rama VI Rd., Bangkok, Thailand 10400

[b]Department of Biochemistry, Faculty of Science, Mahidol University, Rama VI Rd., Bangkok, Thailand 10400

[c]National Center for Genetic Engineering and Biotechnology (BIOTEC), National Science and Technology Development Agency (NSTDA), Yothi office, Rama VI Rd., Bangkok, Thailand 10400

[d]National Center for Genetic Engineering and Biotechnology (BIOTEC), National Science and Technology Development Agency (NSTDA), 113 Thailand Science Park, Phahonyothin Rd., Klong Neung, Klong Luang, Pathum Thani, Thailand 12120

[e]Department of Anatomy, Faculty of Science, Mahidol University, Bangkok, Thailand

[f]Laboratory of Molecular Cell Biology, Center for Excellence in Protein and Enzyme Technology, Faculty of Science, Mahidol University, Bangkok, Thailand 10400

[g]Center of Excellence for Molecular Biology and Genomics of Shrimp, Department of Biochemistry, Faculty of Science, Chulalongkorn University, Bangkok, Thailand 10330

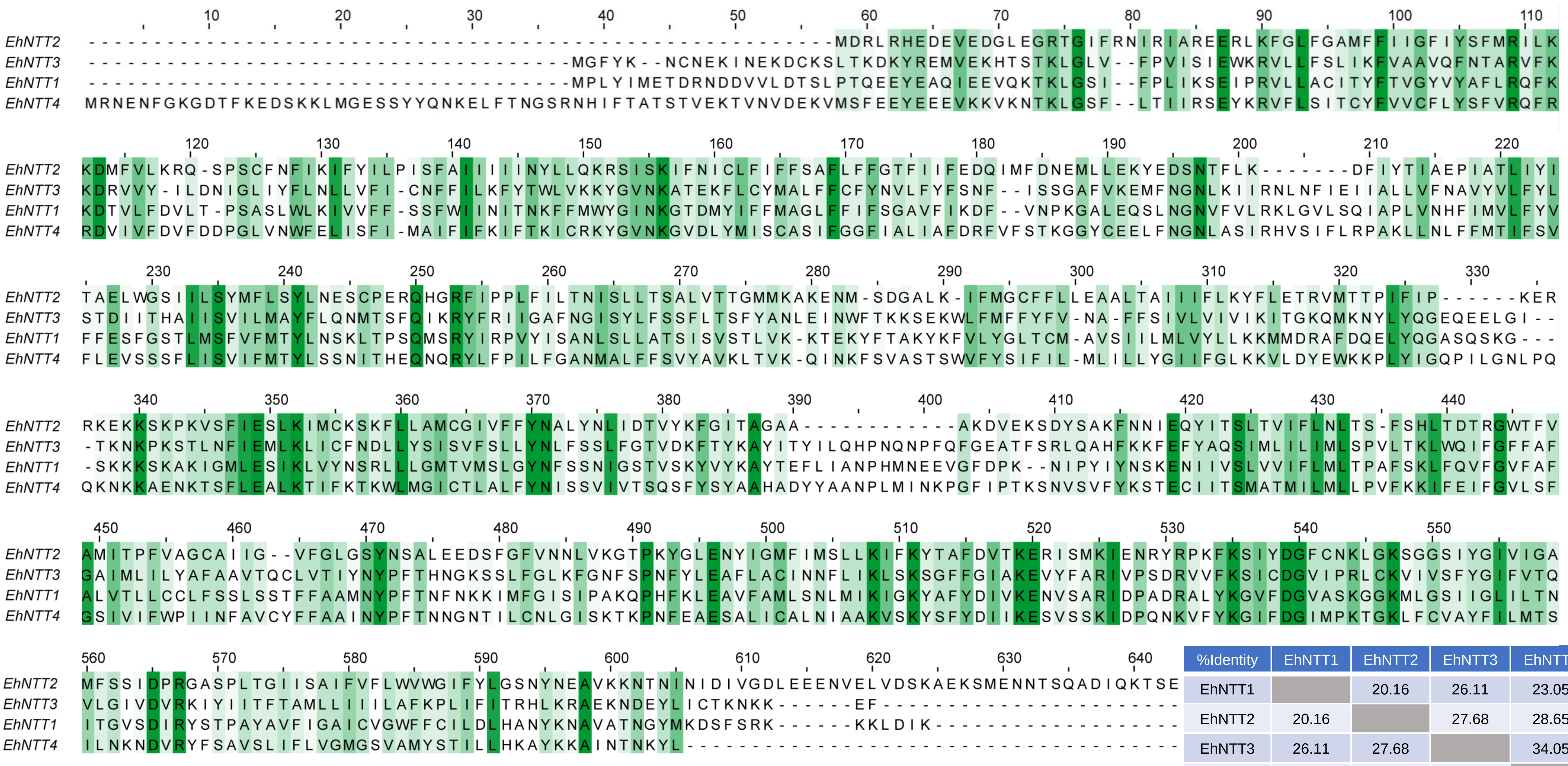

%Identity	EhNTT1	EhNTT2	EhNTT3	EhNTT4
EhNTT1		20.16	26.11	23.05
EhNTT2	20.16		27.68	28.65
EhNTT3	26.11	27.68		34.05
EhNTT4	23.05	28.65	34.05	

Supplementary Figure S1 Amino acid sequence alignment of the four paralogs of EhNTTs. The intensity of the green shading is proportional to the conservation of amino acid residues. The darker shade of green, the more conserved residues (Clamp et al., 2004). A table summarizing percentages of sequence identity is included. The multiple sequence alignment was constructed using the Clustal Omega server (Madeira et al., 2019).

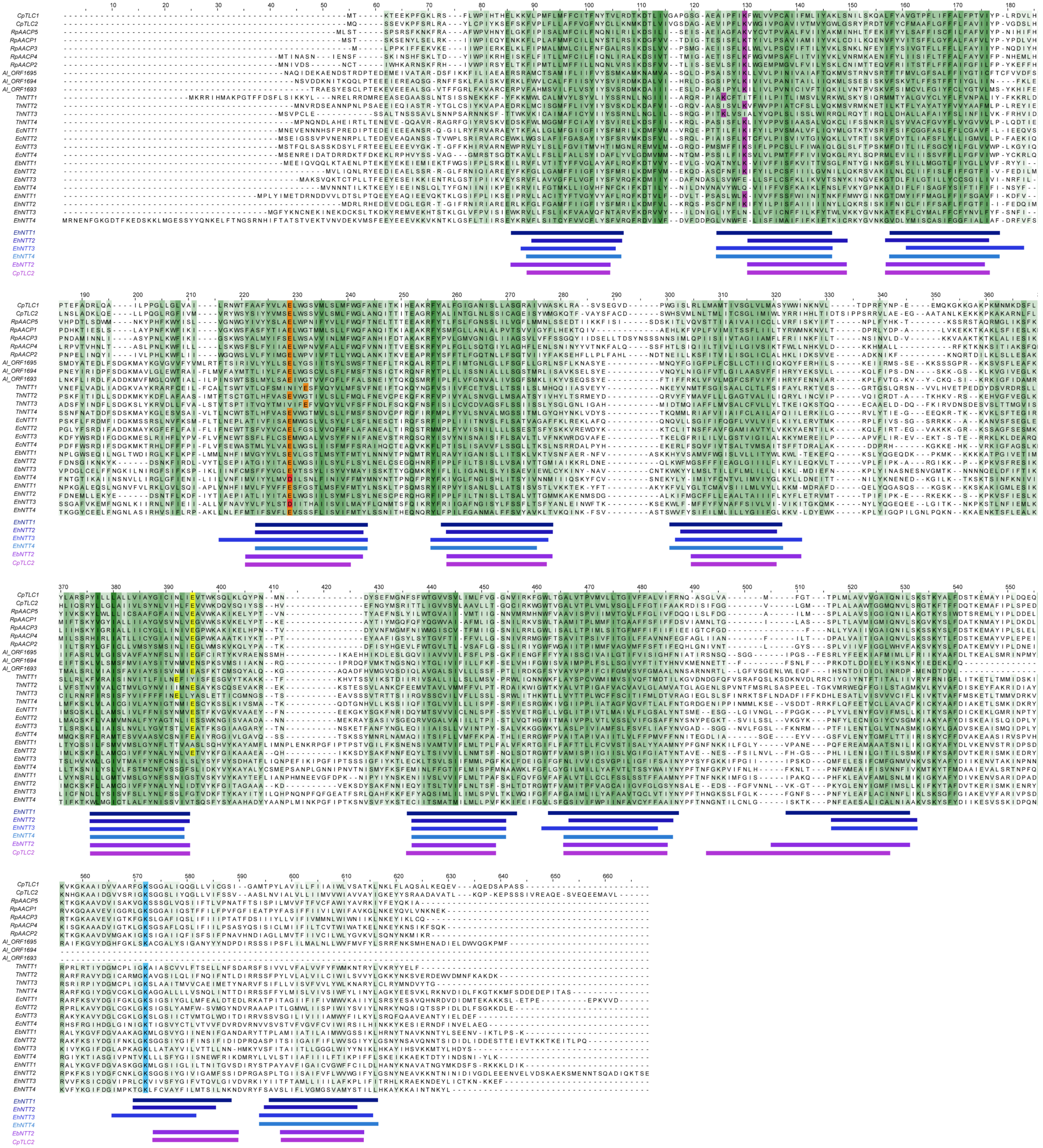

Supplementary Figure S2 Amino acid sequence alignment of representative NTT proteins from bacteria and microsporidian species showing conserved features across the NTT family. The intensity of the green shading is proportional to the conservation of amino acid residues. The darker shade of green, the more conserved residues (Clamp et al., 2004). Four charged residues (K155, E245, E385, and K527) reported to be important for ATP transport in AtAATP1 (Trentmann et al., 2000) were highlighted. Predicted transmembrane domains were shown for EhNTTs, *E. bieneusi* NTT2 (EbNTT2), and *C. pneumoniae* NTT2 (CpNTT2). The multiple sequence alignment was constructed using the Clustal Omega server (Madeira et al., 2019).

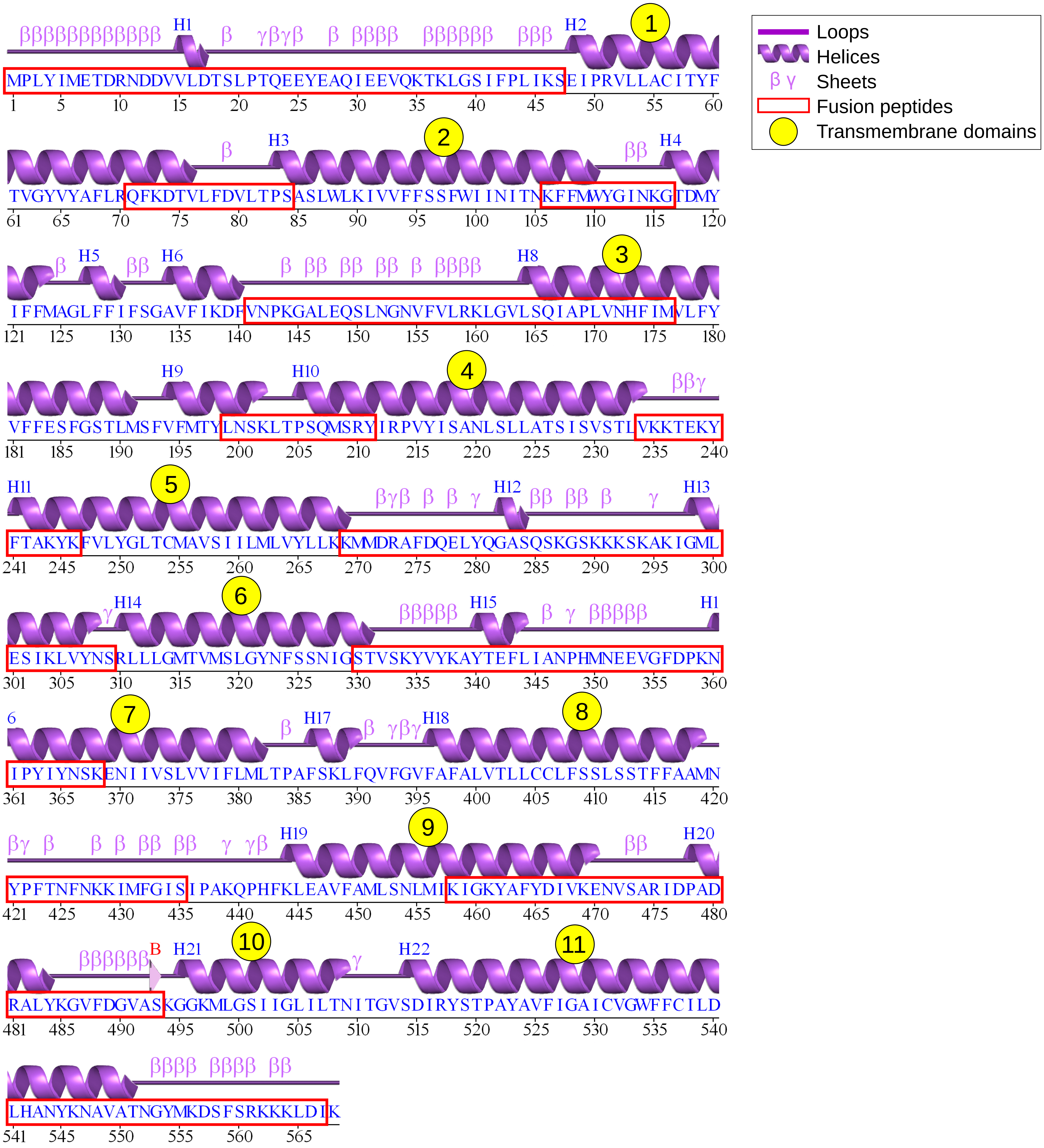

Supplementary Figure S3 Secondary structural prediction of EhNTT1 analyzed by PDBsum (Laskowski et al., 2018). Putative loops, helices, and sheets are demonstrated. Predicted transmembrane helices were specified with numbers (yellow circles). Recombinant EhNTT1 fusion proteins were produced from fusion of peptides proposed to be cytosolic domains (red boxes).

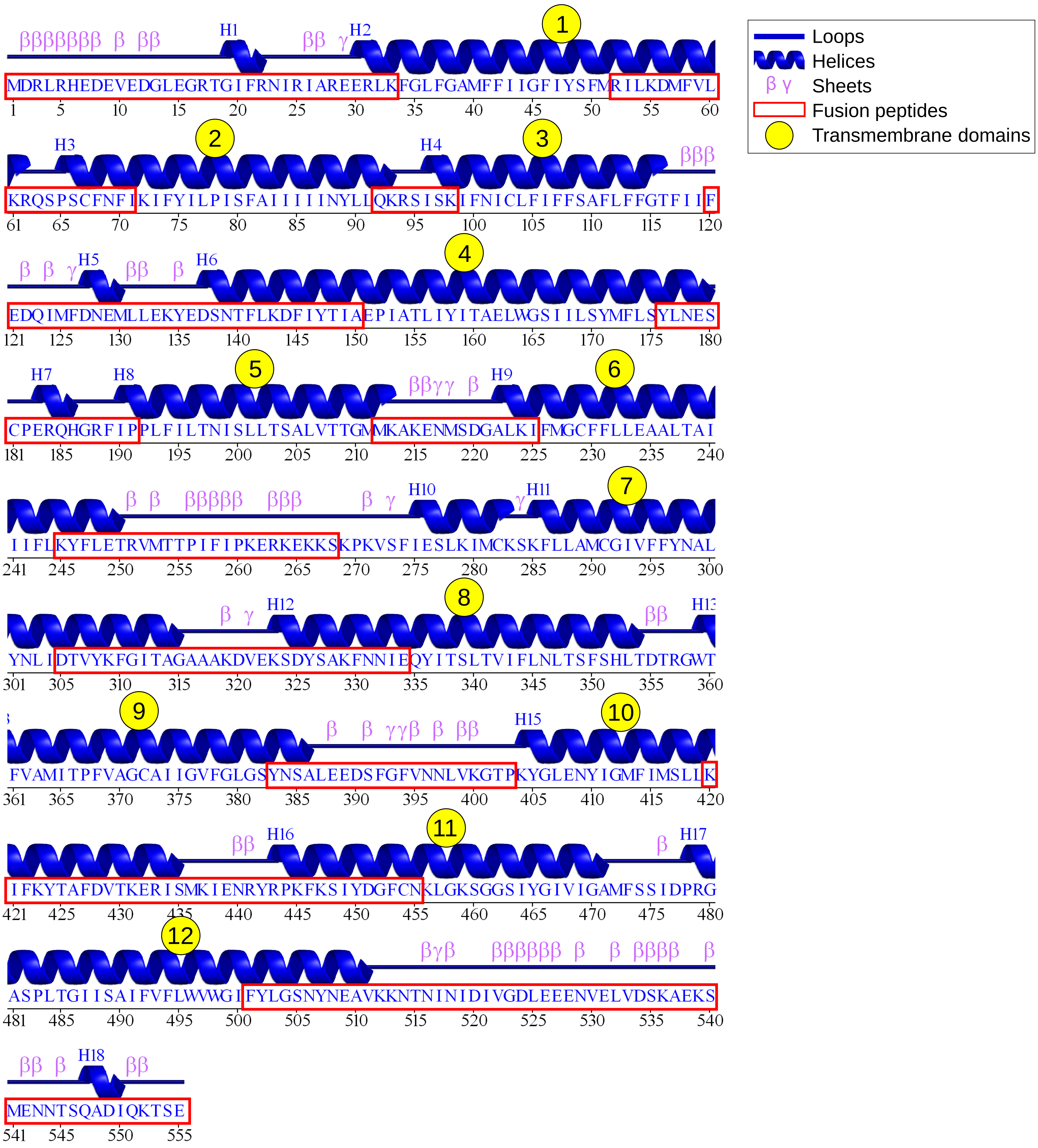

Supplementary Figure S4 Secondary structural prediction of EhNTT2 analyzed by PDBsum (Laskowski et al., 2018). Putative loops, helices, and sheets are demonstrated. Predicted transmembrane helices were specified with numbers (yellow circles). Recombinant EhNTT2 fusion proteins were produced from fusion of peptides proposed to be cytosolic domains (red boxes).

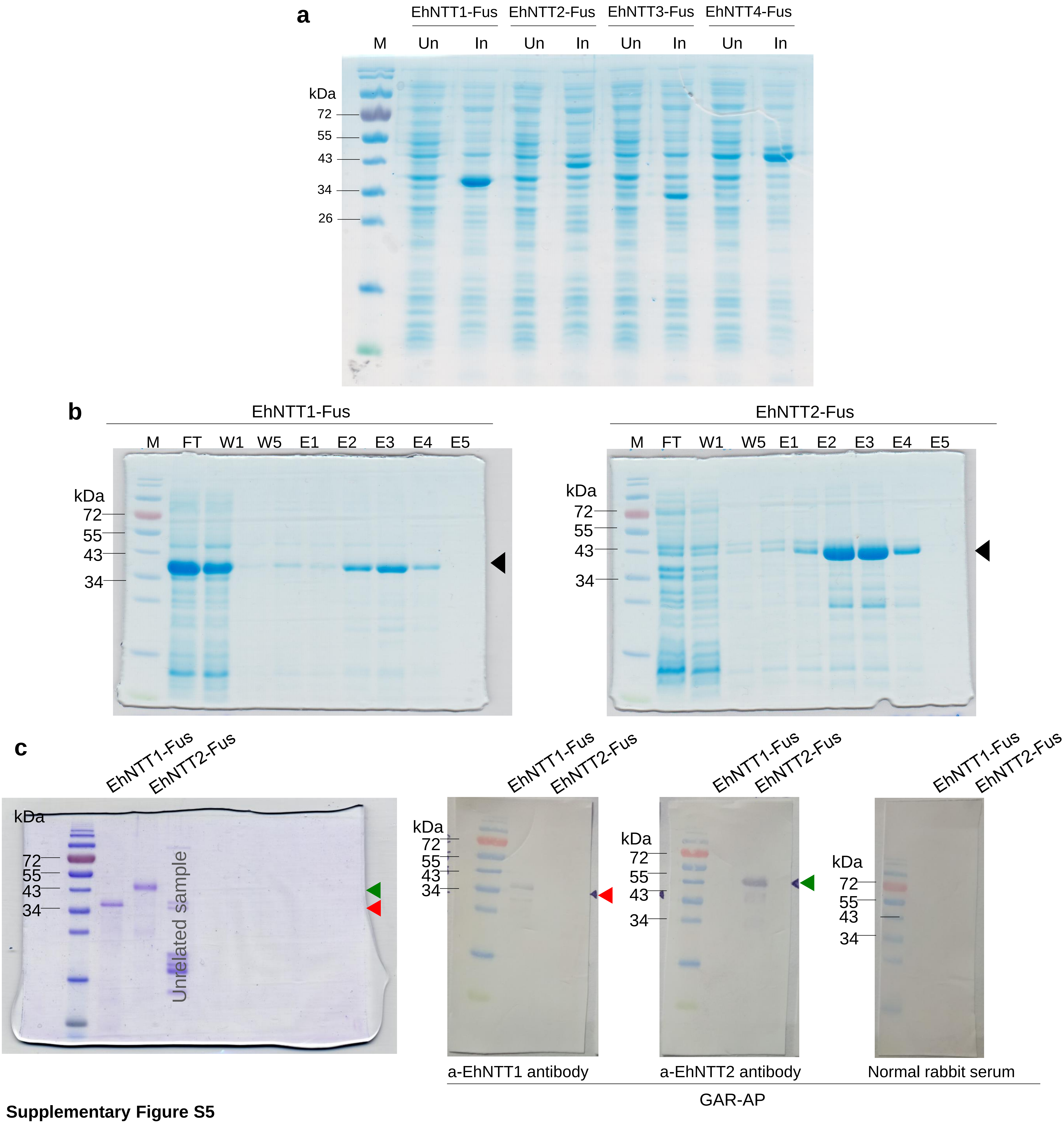

Supplementary Figure S5

Original gel and blot images of Figure 5

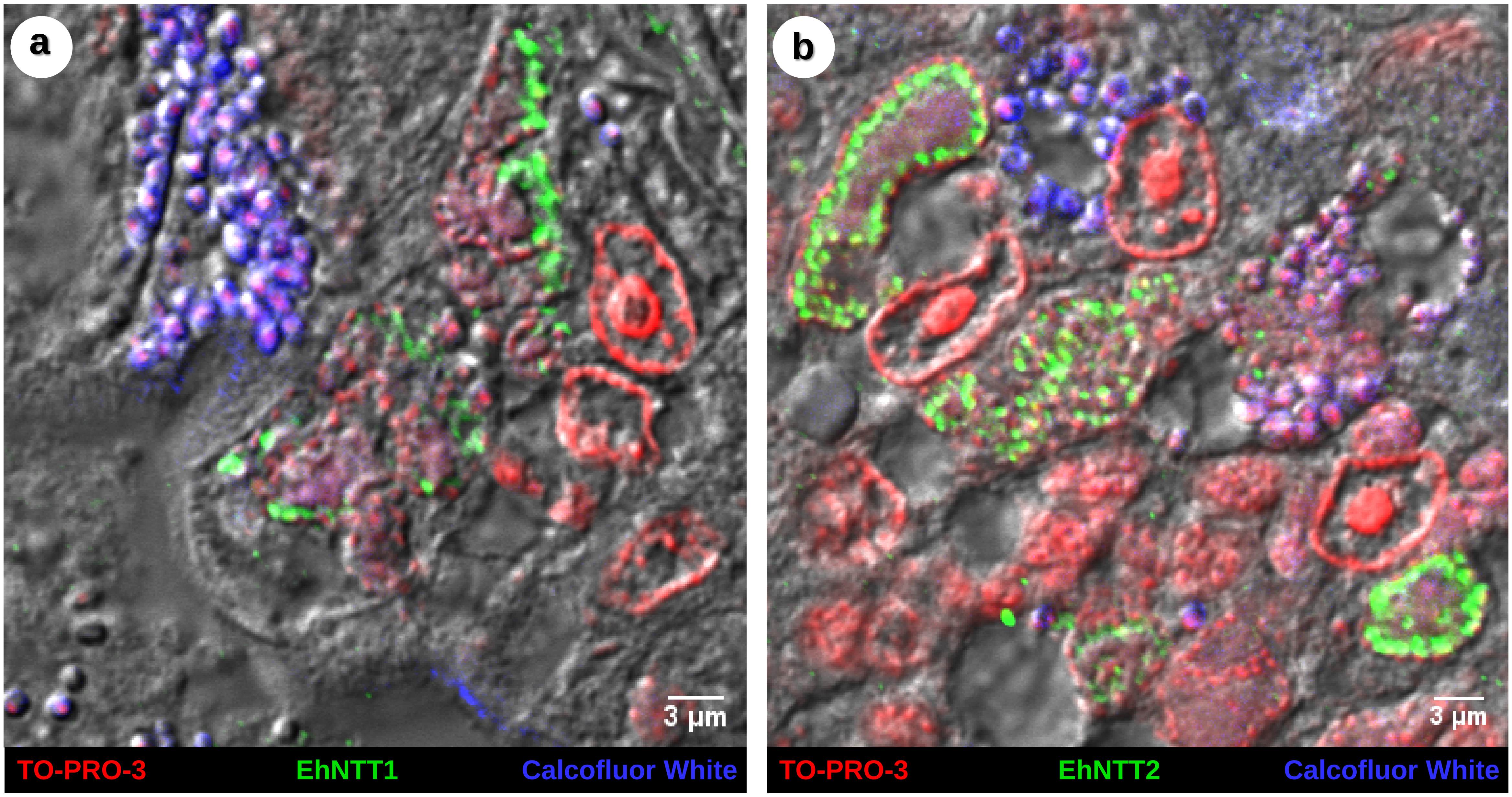

Supplementary Figure S6 Merged images of phase contrast micrographs and three fluorescent channels resulted from IFA of EHP-infected shrimp hepatopancreatic tissue. Red fluorescence labeled large nuclei of *P. vannamei* hepatopancreatic cells (N) and small nuclei of EHP in plasmodia and spore stages. Blue fluorescence labeled chitin on the spore coat. Green fluorescence labeled either EhNTT1 (a) or EhNTT2 (b).

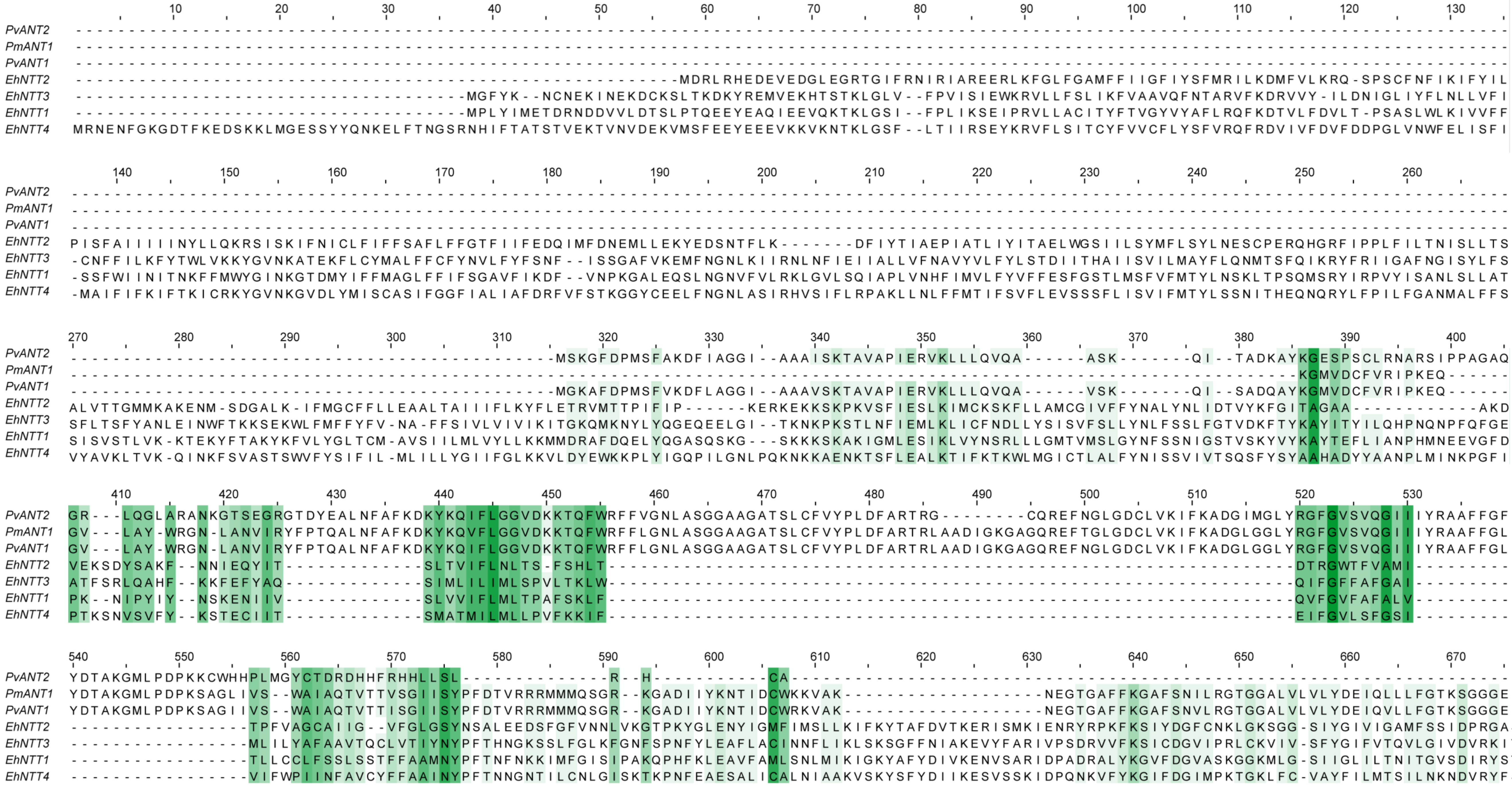

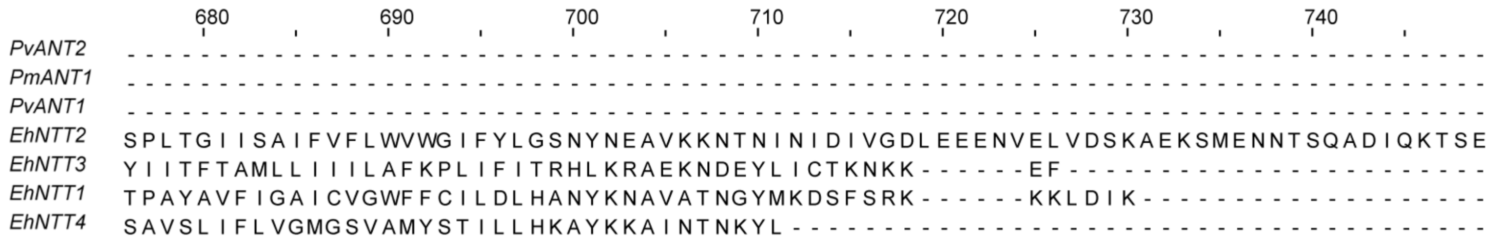

%Identity	PmANT1	PvANT1	PvANT2	EhNTT1	EhNTT2	EhNTT3	EhNTT4
PmANT1		66.67	71.81	13.45	18.57	15.83	11.43
PvANT1	66.67		97.27	13.77	14.00	16.67	18.67
PvANT2	71.81	97.27		14.59	16.75	19.31	15.27
EhNTT1	13.45	13.77	14.59		20.16	26.11	23.05
EhNTT2	18.57	14.00	16.75	20.16		27.68	28.65
EhNTT3	15.83	16.67	19.31	26.11	27.68		34.05
EhNTT4	11.43	18.67	15.27	23.05	28.65	34.05	

Supplementary Figure S7 Amino acid sequence alignment of EhNTT1-4 and the adenine nucleotide translocases from *P. monodon* (PmANT) and *P. vannamei* (PvANT1 and PvANT2). The intensity of the green shading is proportional to the conservation of amino acid residues. The darker shade of green, the more conserved residues (Clamp et al., 2004). A table summarizing percentages of sequence identity is included. The highest and lowest percentage of the identity are highlighted with dark and light green colors, respectively. The multiple sequence alignment was constructed using the Clustal Omega server[54].

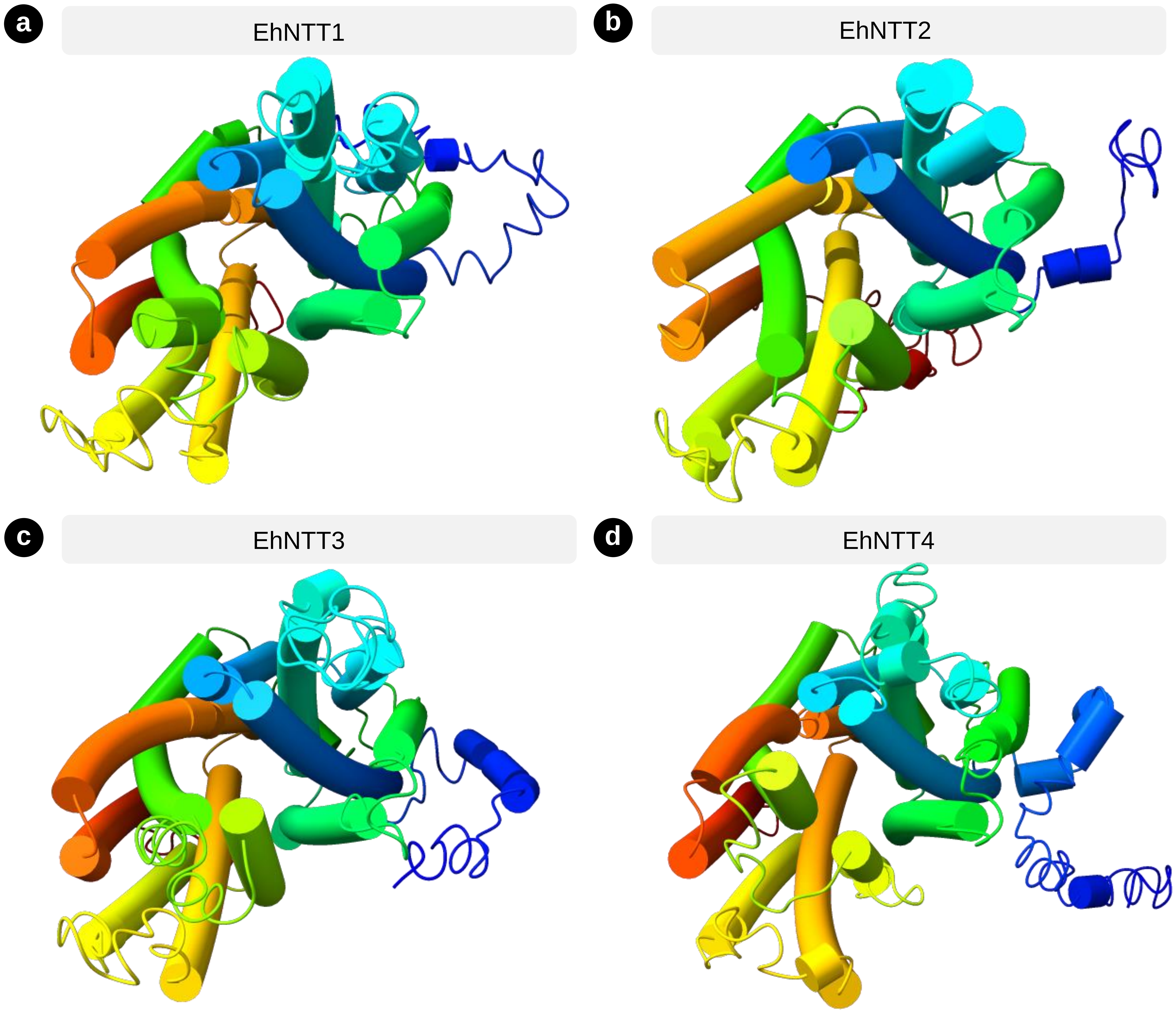

Supplementary Figure S8 Comparison of the predicted secondary structures of EhNTT1-4 showed similar architectures. The protein structures were predicted by I-TASSER Protein Structure Prediction server (Yang et al., 2015). The three-dimensional structures were constructed using ChimeraX (Pettersen et al., 2021). The putative transmembrane helices are depicted as cylinders. The rainbow colors from blue to red represent the helices from N-terminus to C-terminus.

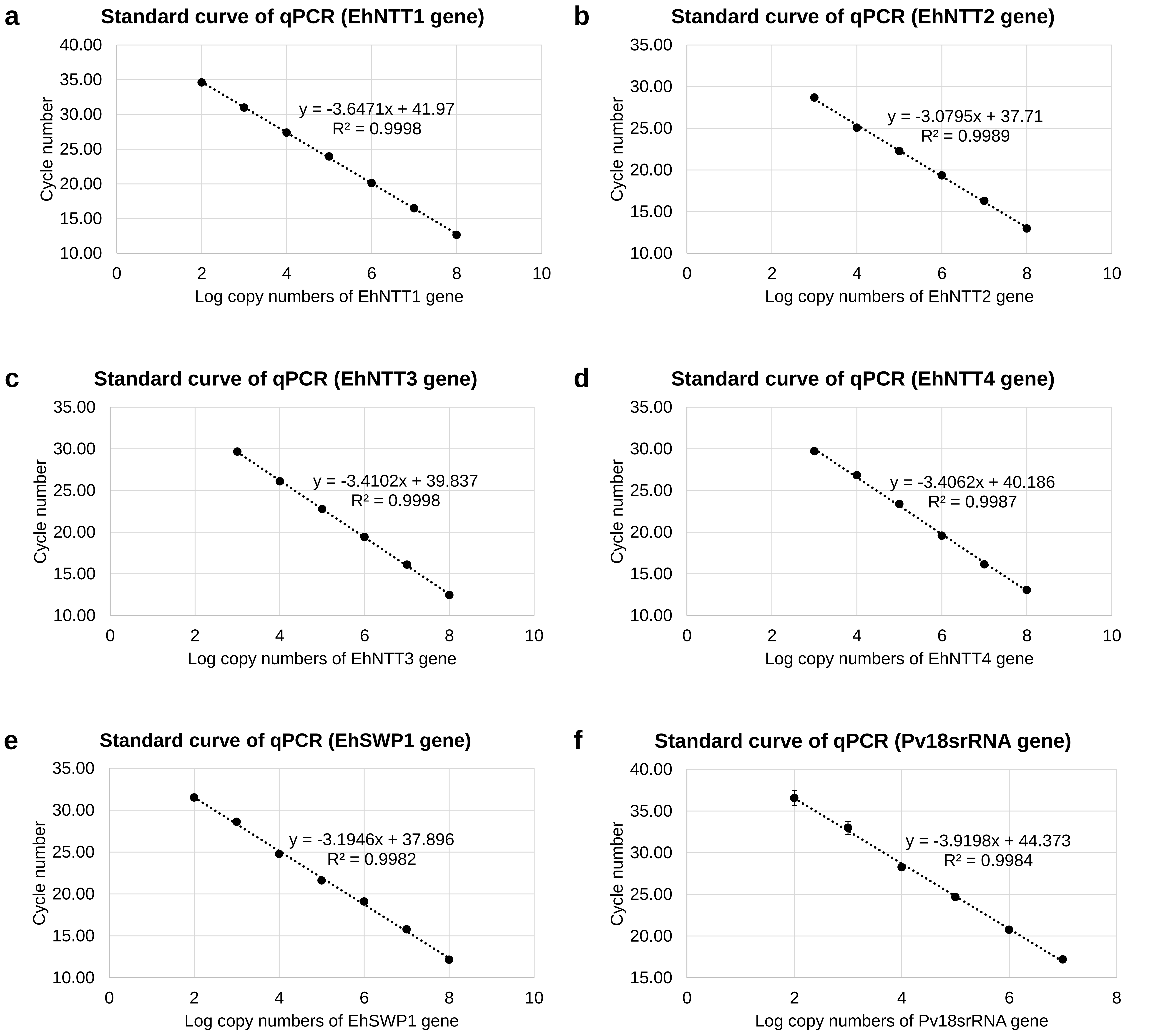

Supplementary Figure S9 Standard curves for gene expression analysis during cohabitation. Linear equations for the calculation of expression level of each gene and qPCR efficiency are shown in the graphs.

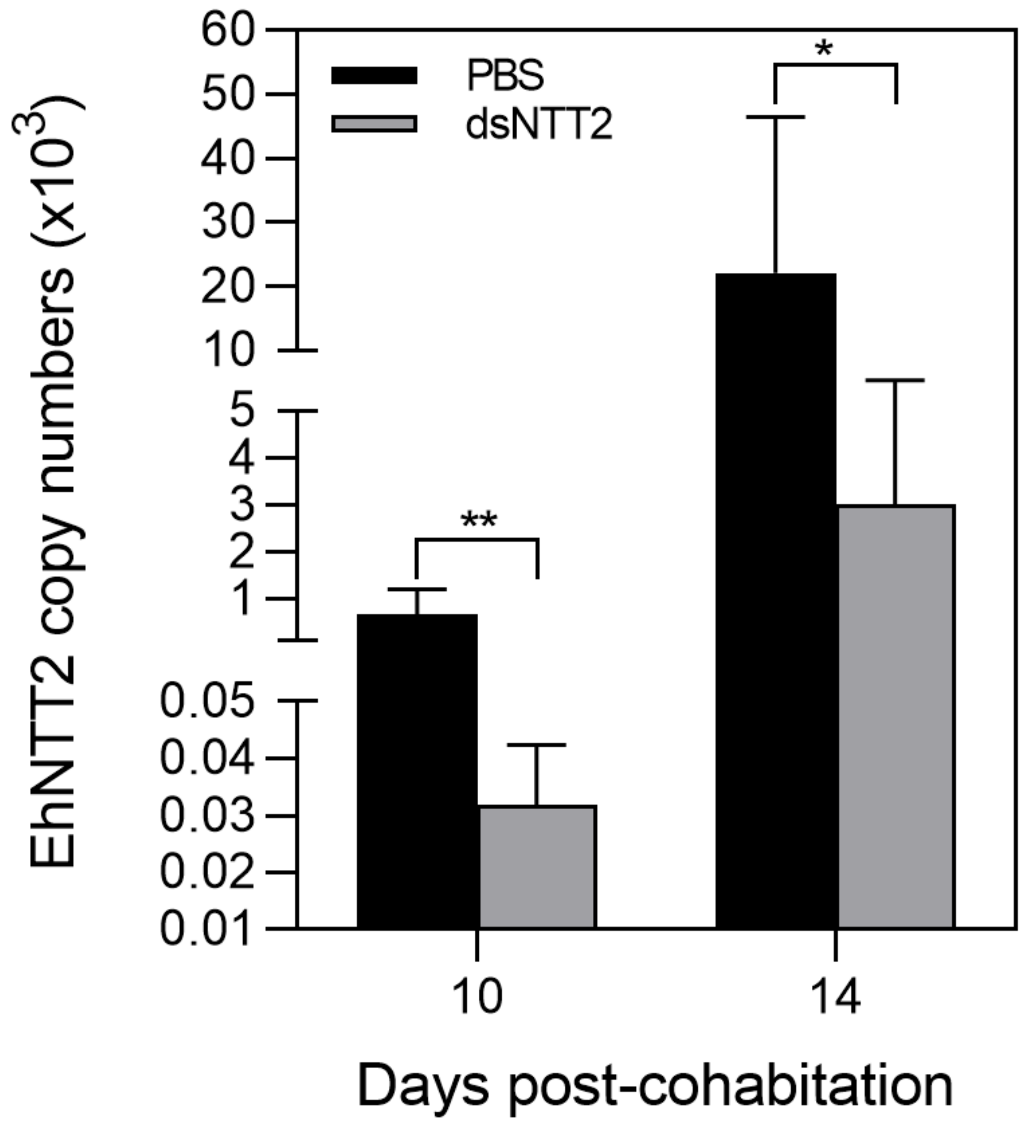

Supplementary Figure S10 EhNTT2 was knocked down upon the injection of EhNTT2-specific dsRNA on Day 10 and 14 post injection. The bar chart illustrating the mean ± standard deviation of copy numbers of the EhNTT2 gene per 100 ng of cDNA from the cohabitated shrimp collected on the day 10 and 14 post-cohabitation, n = 5 per group, except for the group collected on the day 10 post-injected with PBS which n = 6. The shrimp were doubly injected with PBS (black bars) or dsRNA targeting the EhNTT2 gene (gray bars). The asterisks indicate the significant difference from the copy numbers of the EhNTT2 gene between the two groups. The significant levels were determined by the Mann-Whitney test. (* = $p < 0.05$, ** = $p < 0.01$)
